# Supplementary material for: Coordination of humoral immune factors dictates compatibility between Schistosoma mansoni and Biomphalaria glabrata
Source: eLife. 2020 Jan 9;9:e51708. doi: 10.7554/eLife.51708 (PMC6970513; doi:10.7554/eLife.51708)
Supplement: Figure 1—source data 1. — This sequence is derived from GenBank AY028461.1. There are differences in nucleotide sequence but the amino acids match 100%. The differences are due to codon-optimization for expression in insect Sf9 cells. GenBank annotations are used to locate the individual BgFREP3.2 domains: underline, signal peptide; gray shading, IgSF1 domain; double underline, small connecting region; gray shading plus frame, IgSF2 domain; wavy underline, ICR; bold underline, FBG domain; delete line, termination codon. [file elife-51708-fig1-data1.docx]

**>Derived from GenBank AY028461.1 *Biomphalaria glabrata* fibrinogen-related protein 3-2 precursor, gene, complete cds.**

ATGGCTCGTCTCTTCTTGCTCTTCATTCTCTGCGTCTTCGTGGTGTCACTCGCTGGCTCGGAACTCGTCATTGATGTCCAACCCAACGTCATTTCCCCGGAAATCACCCCTCAGCTGGTCATCAACTGCAGCATCACGAACAATGAGGTCCAAACACTGGACCTCATCAAGTCTTTGACCCTGTCAAGGTACAACGAGACTATTCGCGAGTTCGATGAATTGATCGCCCTGGACAGCCTCACCTTGAACCTGAAGCAGTTCGTGCGCTTCAAGTACTCGCAAATCAGTTTCGGCAACCGTTACATCACGCTCATTTTGCACAATCCCACACAGTTCGACGCCCGCATCTACAAGTGCAATGCTACGGGCACAAACTCCGAGGGTGCCAATATCAGCCTGTTCGCCAAGAAGGCTGTGGAGTACGAAACCAACTCTACTGCTCTGATCGAGGAAATTCGCCGTATCAAGAAGGATGAGAACTACTGCAGCTTCAAGAAGGACGATCTGTCTGACTCAAAGCAGCGTTCTAGGGTGTACTTCTCAGGTTCCAGCGATATCATTAAGGAGCGTATCGAACCCCTGACGCTCAAGTGCACATTCCAGGTCCTGAAGACCGACCAAAACGAAACTTCCAGGCTCCAGAGCTTGTACATTCTGCACGAGTCCAAGGGAGTCATCGCCTACGTGAACAAGGATCAGCCAGTGGTCACCTCGCTGCAGGGCAGTAACATCCAAGACGTGGAGGGTGAAATTTACGATAATGCCATCAAGGACTCGTACTTGCAGGTCACTTGGAGTAACCTGAAGCACTCCGAGAGCGGAAAGTACTTCTGCGAAGCTCATAACAAGTACTCGGAGGGCAGGATTGATAAGTCGAGTAATATGCTGACCATCACTGTCGAACGCCCCACCTTCGACGATCTCGTGGAGGCTATGCACAAGTTGTTCACTCAGGTGGACGGTGCCAAGGAGTCCCTGAAGGCTATCAACCAAAACATCAAGAACATCAATAAGGACCTGGATTTCAAGGAACAGAACATTACCTCGATCAAGCAAGAGGTCATCCGCAATCAGAACAATATCCAAATTCTGTCCGAGGATCTCAACATTAAGGAACAGAATCTGACGTCGATCAAGGCCGACCTCAGTACCAAGCAGCAAACTTTCTTGAACATTAAGGAGGATGTGATCCTGAATCAGCAAATCATTCATAAGATCAAGCAGGACCTGAACACCTACCGCCACAATATGTCCAACATTGAGGAACATCTGGAAGTGATCCTCACGAACTTGTCTACAGCTTCAATCAAGGTCAAGAATCAGACGGATGAGGGATCCAAGATGAGCTACCCCCCACGCAAGTCTTGCCGTGATGTGAACTCAACAGACGAGCGTGTGGTCGTGACGTTGACAAGCGGCCTGAAGGTCATGTGCGATACCAAGACTGACGGCGGTGGATGGATCATTTTCCAGAGGCGCATCAACGGTAATGTGGACTTCTACCGTGGATGGAAGGAGTACAGGGATGGCTTCGGTGACTACAACATTGGAGAGTTCTACCTCGGCAACGAAAATATCTACATGTTGACGTCGACAGGCCAGTACAACCTCAGGATCGATTTGAAGTACAAGAATAAGGCCTTCTTCGCTCAATACTCCGGTTTCAAGATTCTGAGCGAGAAGGAAAAGTACAAGCTCAACATCGGAGCCTACTCGGGAAATAGTGGCGACAACTTCTCTTCACACAACAATGCTTTCTTCACCACTTTCGACCGTGATAACGACGAGTACTCCTACAATTGCGCCGTGGATTACACCGGCGCTTGGTGGTACCATTCCAGCTGCCTGAACTGCAATCTCAACGGCAAGTGGGGATCGAGTGACTTCGCCAAGGGTGTCAACTGGTACGACCTGAGCCGTTTCGATTCGTCCGTGTCGTTCACTGAGATGAAGATTAGGGAGATT~~TAA~~

**Figure 1—figure supplement 1. Nucleotide sequence for cloning r*Bg*FREP3.**
