# Supplementary material for: Coordination of humoral immune factors dictates compatibility between Schistosoma mansoni and Biomphalaria glabrata
Source: eLife. 2020 Jan 9;9:e51708. doi: 10.7554/eLife.51708 (PMC6970513; doi:10.7554/eLife.51708)
Supplement: Figure 1—source data 2. — Peptides identified by LC-MS/MS from rBgFREP3 pull-down experiments are highlighted in gray. The GenBank accession numbers of each entry are: BgTEP, ACL00841.1; BgTEP1.1, ADE45332.1; BgTEP1.2, ADE45339.1; BgTEP1.3, ADE45340.1; BgTEP1.4, ADE45341.1 and BgTEP1.5, ADE45333.1. [file elife-51708-fig1-data2.docx]

CLUSTAL O (1.2.4) multiple sequence alignment

*Bg*TEP MRMKLNLILFVFYLVFQECQGGKYFISAPRNVVPGTAYDISVDILKQDIGNVTVEAILQD 60

*Bg*TEP1.1 MRMKLNLILFVFYLVFQECQGGKYFISAPRNVVPGTAYDISVDILKQDIDNVTVEAILQD 60

*Bg*TEP1.2 MRMKLNLILFVFYLVFQECQGGKYFISAPRNVVPGTAYDISVDILKQDIDNVTVEAILQD 60

*Bg*TEP1.3 MRMKLNLILFVFYLVFQECQGGKYFISAPRNVVPGTAYDISVDILKQDIDNVTVGAILQD 60

*Bg*TEP1.4 MRMKLNLILFVFYLVFQECQGGKYFISAPRNVVPGTAYDISVDILKQDIDNVTVEAILQD 60

*Bg*TEP1.5 MRMKLNLILFVFYLVFQECQGGKYFISAPRNVVPGTAYDISVDILKQDIGNVTVEAILQD 60

*************************************************.**** *****

*Bg*TEP YSFSIPEGPKSLLTANGTFSPGVRGTLSMPIDFNLHCSYCRILLKGYNPLQFEQDIFIQI 120

*Bg*TEP1.1 YSFSIPEGPKSLLTANGTFSPGVRGTLSMPIDFNLHCSYCRILLKGYNPLQFEQDIFIQI 120

*Bg*TEP1.2 YSFSIPEGPRSLLTANGTFSPGVRGTLSMPIDFNLHCSYCRILLKGYNPLQFEQDIFIQI 120

*Bg*TEP1.3 YSFSIPEGPKSLLTANGTFSPGVRGTLSMPIDFNLHCSYCRILLKGYNPLQFEQDIFIQI 120

*Bg*TEP1.4 YSFSIPEGPKSLLTANGTFSPGVRGTLSMPIDFNLHCSYCRILLKGYNPLQFEQDIFIQI 120

*Bg*TEP1.5 YSFSIPEGPKSLLTANGTFSPGVRGTLSMPIDFNLHCSYCRILLKGYNPLQFEQDIFIQI 120

*********:**************************************************

*Bg*TEP SSDILSILIQTDKAIYKPKERVNFRILAAYYNLQLYTGTFHYEILDPYDNKINVLSGVSG 180

*Bg*TEP1.1 SSDILSILIQTDKAIYKPKERVNFRILAAYYNLQLYTGTFHYEILDPYDNKINVLSGVSG 180

*Bg*TEP1.2 SSDILSILIQTDKAIYKPKERVNFRILAAYYNLQLYTGTFHYEILDPYDNKINVLSGVSG 180

*Bg*TEP1.3 SSDILSILIQTDKAIYKPKERVNFRILAAYYNLQLYTGTFHYEILDPYDNKINVLSGVSG 180

*Bg*TEP1.4 SSDILSILIQTDKAIYKPKERVNFRILAAYYNLQLYTGTFHYEILDPYDNKINVLSGVSG 180

*Bg*TEP1.5 SSDILSILIQTDKAIYKPKERVNFRILAVYYNLQLYTGTFHYEILDPYDNKINVLSGVSG 180

****************************.*******************************

*Bg*TEP TFGVVEGFFDLSDQPSFGTWKINVRTETVSGEESQLFEVAEYDLPRFQVDVGLPPFALLS 240

*Bg*TEP1.1 TFGVVEGFFDLSDQPSFGTWKINVRTETVSGAESQFFEVAEYDLPRFQVDVGLPPFALLS 240

*Bg*TEP1.2 TFGVVEGFFDLSDQPSFGTWKINVRTETVSGAESQFFEVAEYDLPRFQVDVGLPPFALLS 240

*Bg*TEP1.3 TFGVVEGFFDLSDQPSFGTWKINVRTETVSGAESQFFEVAEYDLPRFQVDVGLPPFALLS 240

*Bg*TEP1.4 TFGVVEGFFDLSDQPSFGTWKINVRTETVSGAESQFFEVAEYDLPRFQVDVGLPPFALLS 240

*Bg*TEP1.5 TFGVVEGFFDLSDQPSFGTWKINVRTETVSGAESQFFEVAEYDLPRFQVDVGLPPFALLS 240

******************************* ***:************************

*Bg*TEP DTTLSGSVEAKYTFGQPVYGLVLLQIGENVDTIDKCNVNRKVTEISFEIKGKENFSVPLE 300

*Bg*TEP1.1 DTTLSGSVEAKYTFGQPVYGLVLLQIGENVDTIDKCNVNRKVTEISFEIKGKGNFSVPLE 300

*Bg*TEP1.2 DTTLSGSVEAKYTFGQPVYGLVLLQIGENVDTIDKCNVNRKVTEISFEIKGKGNFSVPLE 300

*Bg*TEP1.3 DTTLSGSVEAKYTFGQPVYGLVLLQIGENVDTIDKCNVNRKVTEISFEIKGKGNFSVPLE 300

*Bg*TEP1.4 DTTLSGSVEAKYTFGQPVYGLVLLQIGENVDTIDKCNVNRKVTEISFEIKGKGNFSVPLE 300

*Bg*TEP1.5 DTTLSGSVEAKYTFGQPVYGLVLLQIGENVDTIDKCNVNRKVTEISFEIKGKGNFSVPLE 300

**************************************************** *******

*Bg*TEP DIRRSVHLNEKKKIKITAFVTEASTGIKLNGSSVITYYGNRYQIKFLEMTPAVFKPGLQY 360

*Bg*TEP1.1 DIRRSVHLNEKKKIKITAFVTEASTGIKLNGSSVITYYGNRYQIKFLEMTPAVFKPGLQY 360

*Bg*TEP1.2 DIRRSVHLNEKKKIKITAFVTEASTGIKLNGSSVITYYGNRYQIKFLEMTPAVFKPGLQY 360

*Bg*TEP1.3 DIRRSVHLNEKKKIKITAFVTEASTGIKLNGSSVITYYGNRYQIKFLEMTPAVFKPGLQY 360

*Bg*TEP1.4 DIRRSVHLNEKKKIKITAFVTEASTGIKLNGSSVITYYGNRYQIKFLEMTPAVFKPGLQY 360

*Bg*TEP1.5 DIRRSVHLNEKKKIKITAFVIEASTGIKLNGSSVITYYGNRYQIKFLEMTPAVFKPGLQY 360

******************** ***************************************

*Bg*TEP TAYVQVTTPDGLPPTDSNLSLSVYTSVTYQMTVPDQELYSPSSFSGSYPLPGQNMSLPAN 420

*Bg*TEP1.1 TAYVQVTTPDGLPPTDSNLSLSVYTSVTYQMTVPDQELYSPSSFSGSYPLPGQNMSLPAN 420

*Bg*TEP1.2 TAYVQVTTPDGLPPTDSNLSLSVYTSVTYQMTVPDQELYSPSSFSGSYPLPGQNMSLPAN 420

*Bg*TEP1.3 TAYVQVTTPDGLPPTDSNLSLSVYTSVTYQMTVPDQELYSPSSFSGSYPLPGQNMSLPAN 420

*Bg*TEP1.4 TAYVQVTTPDGLPPTDSNLSLSVYTSVTYQMTVPDQELYSPSSFSGSYPLPGQNMSLPAN 420

*Bg*TEP1.5 TAYVQVTTPDGLPPTESNLSLSVYTSVTYQMTVPDQELYSPSSFSGSYPLPGQNMSLPAN 420

***************:********************************************

*Bg*TEP GILSIDIDIPLNATSIDIKVSLNKQTTAEKRISKSYSMSNNYLQLSLLSKLVKAESDVLI 480

*Bg*TEP1.1 GILSIDIDIPLNATSIDIKVSLNKETTAEKRISKSYSMSNNYLQLSLLSKLVKAESDVLI 480

*Bg*TEP1.2 GILSIDIDIPLNATSIDIKVSLNKETTAEKRISKSYSMSNNYLQLSLLSKLVKAESDVLI 480

*Bg*TEP1.3 GILSIDIDIPLNATSIDIKVSLNKETTAEKRISKSYSMSNNYLQLSLLSKLVKAESDVLI 480

*Bg*TEP1.4 GILSIDIDIPLNATSIDIKVSLNKETTAEKRISKSYSMSNNYLQLSLLSKLVKAESDVLI 480

*Bg*TEP1.5 GILSIDIDIPLNATSIDIKVSLNKQTTAEKRISKSYSMSNNYLQLSLLSKLVKAESDVLI 480

************************:***********************************

*Bg*TEP KITSTEAIDSLAYEIRSRSDRVKSGVLELNGQREFNATFKVEPSWAPIAQLLMYYIRRDS 540

*Bg*TEP1.1 KITSTEAIDSLAYEIRSRSDHVKSGVLELSGQREFNATFKVEPSWAPIAQLLMYYIRRDS 540

*Bg*TEP1.2 KITSTEAIDSLAYEIRSRSDHVKSGVLELSGQREFNATFKVEPSWAPIAQLLMYYIRRDS 540

*Bg*TEP1.3 KITSTEAIDSLAYEIRSRSDHVKSGVLELSGQREFNATFKVEPSWAPIAQLLMYYIRRDS 540

*Bg*TEP1.4 KITSTEAIDSLAYEIRSRSDHVKSGVLELSGQREFNATFKVEPSWAPIAQLLMYYIRRDS 540

*Bg*TEP1.5 KITSTEAIDSLAYEIRSRSDHVKSGVLELNGQREFNATFKVGPSWAPIAQLLMYYIRRDS 540

********************:********.*********** ******************

*Bg*TEP NEVVTDSLAFNVEGMFKNKVNVAFKENETDINKNVSLELPADSDSQIYVLAVDXSVLLLK 600

*Bg*TEP1.1 NEVVTDSLAFNVEGMFKNKVNVAFKENETDINKNVSLELSADSDSQIYVLAVDQSVLLLK 600

*Bg*TEP1.2 NEVVTDSLAFNVEGMFKNKVNVAFKENETDINKNVSLELSADSDSQIYVLAVDQSVLLLK 600

*Bg*TEP1.3 NEVVTDSLAFNVEGMFKNKVNVAFKENETDINKNVSLELSADSDSQIYVLAVDQSVLLLK 600

*Bg*TEP1.4 NEVVTDSLAFNVEGMFKNKVNVAFKENETDINKNVSLELSADSDSQIYVLAVDQSVLLLK 600

*Bg*TEP1.5 NEIVTDSLAFNVEGMFENKVNVAFKENETDINKNVSLELSADSDSQIYVLAVDQSVLLLK 600

**:*************:********************** ************* ******

*Bg*TEP TGNDXTPXKVKDSFXSXFHKGAIPTDSNFALSYSGSSIXEVFSNMGLVIATDLNIFAPFR 660

*Bg*TEP1.1 TGNDLTPNKVKDSFISKFHKGAIPTDSNFALSYSGSSINEVFSNMGLVIATDLNIFAPFR 660

*Bg*TEP1.2 TGNDLTPNKVKDSFISKFHKGAIPTDSNFALSYSGSSINEVFSNMGLVIATDLNIFAPFR 660

*Bg*TEP1.3 TGNDLTPNKVKDSFISKFHKGAIPTDSNFALSYSGSSINEVFSNMGLVIATDLNIFAPFR 660

*Bg*TEP1.4 TGNDLTPNKVKDSFISKFHKGAIPTDSNFALSYSGSSINEVFSNMGLVIATDLNIFAPFR 660

*Bg*TEP1.5 TGNDLTPNKVKDSFISKFHKGEVPTDSNFALSYSGSSINEVFSNMGLVIATDLNIFAPFR 660

**** ** ****** * **** :*************** *********************

*Bg*TEP PIALGRFPSSGFDRQXMMGAPXAMSFXDDXAMXSASFEMDVTTSTKPVERVRSFFPESWL 720

*Bg*TEP1.1 PIALGRFPSSGFDRQGMMGAPMAMSFRDDNAMESASFEMDVATSTKPVERVRSFFPESWL 720

*Bg*TEP1.2 PIALGRFPSSGFDRQGMMGAPMAMSFRDDNAMESASFEMDVATSTKPVERVRSFFPESWL 720

*Bg*TEP1.3 PIALGRFPSSGFDRQGMMGAPMAMSFRDDNAMESASFEMDVATSTKPVERVRSFFPESWL 720

*Bg*TEP1.4 PIALGRFPSSGFDRQGMMGAPMAMSFRDDNAMESASFEMDVATSTKPVERVRSFFPESWL 720

*Bg*TEP1.5 PIALGRFPSSGFDRQGMMGAPMAMSFRDDNAMESASFEMDVATSTKPVERVRSFFPESWL 720

*************** ***** **** ** ** ********:******************

*Bg*TEP WTSVKSINGHATLTTTVPDTITSWIGSAFATNSDTGLGVAPTTSKLPGFRPFFGSLTYPR 780

*Bg*TEP1.1 WTSVKSINGHATLTTTVPDTITSWIVSAFATNSDTGLGVAPTTSKLRVFRPFFVSLTYPR 780

*Bg*TEP1.2 WTSVKSINGHATLTTTVPDTITSWIVSAFATNSDTGLGVAPTTSKLRVFRPFFVSLTYPR 780

*Bg*TEP1.3 WTSVKSINGHATLTTTVPDTITSWIVSAFATNSDTGLGVAPTTSKLRVFRPFFVSLTYPR 780

*Bg*TEP1.4 WTSVKSINGHATLTTTVPDTITSWIVSAFATNSDTGLGVAPTTSKLRVFRPFFVSLTYPR 780

*Bg*TEP1.5 WTSVKSINGHATLTTTVPDTITSWIVSAFATNSDTGLGVAPTTSKLRVFRPFFVSLTYPR 780

************************* ******************** ***** ******

*Bg*TEP SVTRNEQFIVQATVFNYLPVDLMVTVSLKENPFLTPVTPGPGNQASNIQVRANEQRTVYF 840

*Bg*TEP1.1 SVTRNEQFIVQATVFNYLPVDLMVTVSLKENPFLTPITPGPGNQASNIQVRANEQGIVYF 840

*Bg*TEP1.2 SVTRNEQFIVQATVFNYLPVDLMVTVSLKENPFLTPITPGPGNQASNIQVRANEQGIVYF 840

*Bg*TEP1.3 SVTRNEQFIVQATVFNYLPVDLMVTVSLKENPFLTPITPGPGNQASNIQVRANEQGIVYF 840

*Bg*TEP1.4 SVTRNEQFIVQATVFNYLPVDLMVTVSLKENPFLTPITPGPGNQASNIQVRANEQGIVYF 840

*Bg*TEP1.5 SVTRNEQFIVQATVFNYLPVDLMVTVSLKENPFLTPITPGPGNQASNIQVRANEQGIVYF 840

************************************:****************** ***

*Bg*TEP SLSALIVGALDIEVSARSNMAADAIVRQILVRHEGAPVVYNNPILISLSNNQSTFEKNIA 900

*Bg*TEP1.1 SLSALTVGSLDIEVSARSNMAADAIVRQILIKHEGAPVVYNNPILINLSNNQSTFEKNIA 900

*Bg*TEP1.2 SLSALTVGSLDIEVSARSNMAADAIVRQILIKHEGAPVVYNNPILINLSNNQSTFEKNIA 900

*Bg*TEP1.3 SLSALTVGSLDIEVSARSNMAADAIVRQILIKHEGAPVVYNNPILINLSNNQSTFEKNIA 900

*Bg*TEP1.4 SLSAPTVGSLDIEVSARSNMAADAIVRQILIKHEGAPVVYNNPILINLSNNQSTFEKNIA 900

*Bg*TEP1.5 SLSALTVGSLDTEVSARSNMAADAIVRQILIKHEGAPVVYNNPILINLSNNQSTFEKNIA 900

**** **:** ******************::**************.*************

*Bg*TEP FTLPDSLVPESHRIRVKVTGDLIGSTVQSLTSLLTLPTGCGEQSLVKFTPNIHIGRYLKA 960

*Bg*TEP1.1 FTLPDSLVPESQRIRVKVTGDLIGSTVQSLTSLLTLPTGCGEQSLVKFTPNIHIGRYLKA 960

*Bg*TEP1.2 FTLPDSLVPESQRIRVKVTGDLIGSTVQSLTSLLTLPTGCGEQSLVKFTPNIHIGRYLKA 960

*Bg*TEP1.3 FTLPDSLVPESQRIRVKVTGDLIGSTVQSLTSLLTLPTGCGEQSLVKFTPNIHIGRYLKA 960

*Bg*TEP1.4 FTLPDSLVPESQRIRVKVTGDLIGSTVQSLTSLLTLPTGCGEQSLVKFTPNIHIGRYLKA 960

*Bg*TEP1.5 FTLPDSLVPESQRIRVKVTGDLIGSTVQSLTSLLTLPTGCGEQSLVKFTPNIHIGRYLKA 960

***********:************************************************

*Bg*TEP TNQLSKELNKKIIDLLNNGYQRQLTYKRYDNGFSAFGNYDLSSSTWLTALVVTSFAEAQE 1020

*Bg*TEP1.1 TNQLSEELNKKIIDLLNDGYQRQLTYKRYDNGFSAFGNYDISSSTWLTALVVTSFAEAQE 1020

*Bg*TEP1.2 TNQLSEELNKKIIDLLNDGYQRQLTYKRYDNGFSAFGNYDISSSTWLTALVVTSFAEAQE 1020

*Bg*TEP1.3 TNQLSEELNKKIIDLLNDGYQRQLTYKRYDNGFSAFGNYDISSSTWLTALVVTSFAEAQE 1020

*Bg*TEP1.4 TNQLSEELNKKIIDLLNDGYQRQLTYKRYDNGFSAFGNYDISSSTWLTALVVTSFAEAQE 1020

*Bg*TEP1.5 TNQLSEELNKKIIDLLNDGYQRQLTYKRYDNGFSAFGNYDISSSTWLTALVVTSFAEAQE 1020

*****:***********:**********************:*******************

*Bg*TEP FIFVDKEIILKASMLLIDRQNLDGSFNEFGKVLDRNTQGT-TAGPALTAFVLVALLKAKE 1079

*Bg*TEP1.1 FIFVDKEIILKASMLLIDRQNIDGSFNEFGKVLDRNTQGT-TAGPALTAFVLVALLKAKE 1079

*Bg*TEP1.2 FIFVDKEIILKASMLLIDRQNIDGSFNEFGKVLDRNTQGT-TAGPALTAFVLVALLKAKE 1079

*Bg*TEP1.3 FIFVDKEIILKASMLLIDRQNIDGSFNEFGKVLDRNTQGT-TAGPALTAFVLVALLKAKE 1079

*Bg*TEP1.4 FIFVDKEIILKASMLLIDRQNIDGSFNEFGKVLDRNTQGT-TAGPALTAFVLVALLKAKE 1079

*Bg*TEP1.5 FIFVDKEIILKASMLLIDRQNIDGSFNEFGKVLDRNTQGTTTAGPALTAFVLVALLKAKE 1080

*********************:****************** *******************

*Bg*TEP LADVQYCKNNNKCRYYLLGNATLNATRNLERLMLADSIDDQFSLAVTSYALAEAKSQLAQ 1139

*Bg*TEP1.1 LADVQDCKNNNKCRYYLLGNATLNATRNLERLMLADSIDDQFSLAVASYAFAEAKSQLAQ 1139

*Bg*TEP1.2 LADVQDCKNNNKCRYYLLGNATLNATRNLERLMLADSIDDQFSLAVASYAFAEAKSQLAQ 1139

*Bg*TEP1.3 LADVQDCKNNNKCRYYLLGNATLNATRNLERLMLADSIDDQFSLAVASYAFAEAKSQLAQ 1139

*Bg*TEP1.4 LADVQDCKNNNKCRYYLLGNATLNATRNLERLMLADSIDDQFSLAVASYAFAEAKSQLAQ 1139

*Bg*TEP1.5 LADVQDCKNNNKCRYYLLGNATLNATRNLERLMLADSIDDQFSLAVASYALAEAKSQLAQ 1140

***** ****************************************:***:*********

*Bg*TEP STFEKLLTFVKQEGGLEYRSANSTVNNEELNRFINWRPPRLQARPIDILITSYAILTYSP 1199

*Bg*TEP1.1 STFEKLLTFVKQEGGLEYWSANSTVNNEELNRFINWRPPRLQARPIDILITSYAILTYSS 1199

*Bg*TEP1.2 STFEKLLTFVKQEGGLEYWSANSTVNNEELNRFINWRPPRLQARPIDILITSYAILTYSS 1199

*Bg*TEP1.3 STFEKLLTFVKQEGGLEYWSANSTVNNEELNRFINWRPPRLQARPIDILITSYAILTYSS 1199

*Bg*TEP1.4 STFEKLLTFVKQEGGLEYWSANSTVNNEELNRFINWRPPRLQARPIDILITSYAILTYSS 1199

*Bg*TEP1.5 STFEKLLTFVKQEGGLEYWSANSTVNNEELNRFINWRPPRLQARPIDILITSYAILTYSS 1200

****************** ****************************************

*Bg*TEP LGRLDEALPSVRWLTLQKNAQGGFVSTQDTVVGLQALSTYGSKSFRPDTNITIYVSDMNT 1259

*Bg*TEP1.1 LGRLDEALPSVRWLTLQKNAQGGFVSTQDTVVGLQALSSYGSKSFRPDTNITIYVSDMNT 1259

*Bg*TEP1.2 LGRLDEALPSVRWLTLQKNAQGGFVSTQDTVVGLQALSSYGSKSFRPDTNITIYVSDMNT 1259

*Bg*TEP1.3 LGRLDEALPSVRWLTLQKNAQGGFVSTQDTVVGLQALSSYGSKSFRPDTNITIYVSDMNT 1259

*Bg*TEP1.4 LGRLDEALPSVRWLTLQKNAQGGFVSTQDTVVGLQALSSYGSKSFRPDTNITIYVSDMNT 1259

*Bg*TEP1.5 LGRLDEALPSVRWLTLQKNAQGGFVSTQDTVVGLQALSFYGSKSFRPDTNITIYVSDMNT 1260

************************************** *********************

*Bg*TEP HLTMNVKSDNALSLQIQEIQSNSQDFSITASGSGLALLDIEYSFNVLKELSKPVFDVNTV 1319

*Bg*TEP1.1 HLTMNVNSENALSLQIQEIQSNSQDFSITASGSGLALLDIEYSFNVLKELSKPVFDVNTV 1319

*Bg*TEP1.2 HLTMNVNSENALSLQIQEIQSNSQDFSITASGSGLALLDIEYSFNVLKELSKPVFDVNTV 1319

*Bg*TEP1.3 HLTMNVNSENALSLQIQEIQSNSQDFSITASGSGLALLDIEYSFNVLKELSKPVFDVNTV 1319

*Bg*TEP1.4 HLTMNVNSENALSLQIQEIQSNSQDFSITASGSGLALLDIEYSFNVLKELSKPVFDVNTV 1319

*Bg*TEP1.5 HLTMNVNSENALSLQIQEIQSNSQDFSITASGSGLALLDIEYSFNVLKELSKPVFDANTV 1320

******:*:***********************************************.***

*Bg*TEP LLDDKLDSFNIMVCTKFLLKHDTGMVVQEVSIPSGFVPDLSTLGQVAGVKRSERKGSIVA 1379

*Bg*TEP1.1 LLDDKLDSFNIMVCTKFLMKHDTGMVVQELSIPSGFVPDLSTLGQVAGVKRSERKGSIVA 1379

*Bg*TEP1.2 LLDDKLDSFNIMVCTKFLMKHDTGMVVQELSIPSGFVPDLSTLGQVAGVKRSERKGSIVA 1379

*Bg*TEP1.3 LLDDKLDSFNIMVCTKFLMKHDTGMVVQELSIPSGFVPDLSTLGQVAGVKRSERKGSIVA 1379

*Bg*TEP1.4 LLDDKLDSFNIMVCTKFLMKHDTGMVVQELSIPSGFVPDLSTLGQVAGVKRSERKGSIVA 1379

*Bg*TEP1.5 LLDDKLDSFNIMVCTKFLMKHDTGMVVQELSIPSGFVPDLSTLGQVAGVKRSERKGSIVA 1380

******************:**********:******************************

*Bg*TEP IYFDKISGSSLCYSIVMTREAKVAKSQKSYVRTYDYYEPANQATVFYQPRTLRDSTVCDV 1439

*Bg*TEP1.1 IYFDKISGSSLCYSIVMTREAKVAKSQKSYVRTYDYYEPANQATVFYQPRTLRDSTVCDV 1439

*Bg*TEP1.2 IYFDKISGSSLCYSIVMTREAKVAKSQKSYVRTYDYYEPANQATVFYQPRTLRDSTVCDV 1439

*Bg*TEP1.3 IYFDKISGSSLCYSIVMTREAKVAKSQKSYVRTYDYYEPANQATVFYQPRTLRDSTVCDV 1439

*Bg*TEP1.4 IYFDKISGSSLCYSIVMTREAKVAKSQKSYVRTYDYYEPANQATVFYQPRTLRDSTVCDV 1439

*Bg*TEP1.5 IYFDKISGSSLCYSIVMTREAKVAKSQKSYVRTYDYYEPANQATVFYQPRTLRDSTVCDV 1440

************************************************************

*Bg*TEP CLNCCP 1445

*Bg*TEP1.1 CLNCCP 1445

*Bg*TEP1.2 CLNCCP 1445

*Bg*TEP1.3 CLNCCP 1445

*Bg*TEP1.4 CLNCCP 1445

*Bg*TEP1.5 CLNCCP 1446

******

**Figure 1—figure supplement 2. The peptides identified by LC-MS/MS were distributed over the full-length *Bg*TEP.**
