# Supplementary material for: Coordination of humoral immune factors dictates compatibility between Schistosoma mansoni and Biomphalaria glabrata
Source: eLife. 2020 Jan 9;9:e51708. doi: 10.7554/eLife.51708 (PMC6970513; doi:10.7554/eLife.51708)
Supplement: Figure 1—source data 3. — (A) Alignment analysis of AGG38744.1 and A0A182YTN9. (B) Alignment analysis of AGG38744.1 and A0A182YTZ4. (C) Multiple sequence alignment of AGG38744.1, A0A182YTN9 and A0A182YTZ4. The identified peptides in A, B and C are highlighted in gray. The yellow squares and red plus signs in A and B represent sites with differing amino acids. The red frame in A, B and C representing differences in peptide sequences that distinguish two Biomphalysin variants (A0A182YTN9 and A0A182YTZ4). [file elife-51708-fig1-data3.docx]

**A:**

**AGG38744.1 VS A0A182YTN9** Score:1143 bits(2957), Expect:0.0, Method:Compositional matrix adjust.,Identities:538/572(**94%**), Positives:555/572(97%), Gaps:0/572(0%)

AGG38744.1 MFLQIFVAVTLVQYVSSQCTYSSWWYSFDTPGQSKCNEINSYINALDRNDVNWADDALSN 60

M +Q A TL+QYVSSQCTYSSWWYSFDTPGQSKCNEINSYINALDRNDVNWADDALSN

A0A182YTN9 MLVQFLFAATLLQYVSSQCTYSSWWYSFDTPGQSKCNEINSYINALDRNDVNWADDALSN 60

AGG38744.1 LEGVQCCRPPAPWNNVEQQVVYEDWTATLDSDYTWAFCRVGYFLQGLYRSDTGWPRFKGY 120

LEGVQCCRPPAPWNNVEQQVVYEDWTATLDSDYTWAFCRVGYFLQGLYRSDTGWPRFKGY

A0A182YTN9 LEGVQCCRPPAPWNNVEQQVVYEDWTATLDSDYTWAFCRVGYFLQGLYRSDTGWPRFKGY 120

AGG38744.1 LFNLESARCTKPANHPLNYGTCQDIDVSSCMGRKGQCSCPGGYFLTGLYRADGDDLYFLK 180

LFNLESARCTKPANHPLNYG CQDIDVSSCMGRKGQCSCPGGYFLTGLYRADGDDLYFLK

A0A182YTN9 LFNLESARCTKPANHPLNYGNCQDIDVSSCMGRKGQCSCPGGYFLTGLYRADGDDLYFLK 180

AGG38744.1 KIRCCTPAAKPLEMDEKSKIQTRIMDTTLWNMATLAHYMGYGWCYGCHGLAVGEDFTRNG 240

KIRCCTPAAKPLEMDEKSKIQTRIMDTTLWNMATLAHY+GYGWCYGC G+AVGEDFTRNG

A0A182YTN9 KIRCCTPAAKPLEMDEKSKIQTRIMDTTLWNMATLAHYLGYGWCYGCRGVAVGEDFTRNG 240

AGG38744.1 FTWAADTRTFWGKWCEGDKNGERLNLVFGDWGFAVKEIIYGKSVIEDLQAESVDSGVLYN 300

FTWAADTR+FWGKWCEGDKNGERLNLVFGDWGFAVKEIIYGKSVIEDLQAESVDSGVLYN

A0A182YTN9 FTWAADTRSFWGKWCEGDKNGERLNLVFGDWGFAVKEIIYGKSVIEDLQAESVDSGVLYN 300

AGG38744.1 RASSPVTESIERSKTIQETITHSTTSTFTNSHGLGVELEFEIASVKGKASYKTRFEYSTS 360

RASSPVTESI+R+KTI+ET+THSTTSTFTNSH LG+EL FEIASV GKASY T+FEYS +

A0A182YTN9 RASSPVTESIDRTKTIEETVTHSTTSTFTNSHELGIELNFEIASVSGKASYTTKFEYSKA 360

AGG38744.1 TTNSKSISETQGFTKQSSITLGPMEGAKYEVIMSKSRTTVPYTAIITTKFSTEMKGFLRW 420

TTN KSIS+T GFTK+SSITLGPMEGAKYE+IMSKSRTTVPYTAIITTKFSTEMKGFLRW

A0A182YTN9 TTNEKSISQTAGFTKKSSITLGPMEGAKYEIIMSKSRTTVPYTAIITTKFSTEMKGFLRW 420

AGG38744.1 EDGNGNFHQDYRTNSGRPTFNYRFGDSSVPFYKALKKQSDNNEGVWMWGMLFQKFPDARR 480

EDGNGNFHQDYRTNSGRPT+NYRFGDSSVPFYKALKKQSDNNEGVWMWGMLFQKFPDARR

A0A182YTN9 EDGNGNFHQDYRTNSGRPTYNYRFGDSSVPFYKALKKQSDNNEGVWMWGMLFQKFPDARR 480

AGG38744.1 VTNRLTDETQYQFTLAGKLEKVEGTSVNVKWEKMKLNRRDVSGNDEPGSNITTYIAASGP 540

V NRLTDETQYQFTL GKLEKVEGTSVNVKWEK+KLNRRDVSGND PGSNITTYIAASGP

A0A182YTN9 VINRLTDETQYQFTLTGKLEKVEGTSVNVKWEKIKLNRRDVSGNDAPGSNITTYIAASGP 540

AGG38744.1 ADKPAVVEYPKVNLNNKEPFKPIEIPVTEVKV 572

ADKPAVVEYPKVNLNNKEPFKPIEIPVTEVKV

A0A182YTN9 ADKPAVVEYPKVNLNNKEPFKPIEIPVTEVKV 572

**B:**

**AGG38744.1 VS A0A182YTZ4** Score:1184 bits(3064), Expect:0.0, Method:Compositional matrix adjust.,Identities:565/572(**99%**), Positives:567/572(99%), Gaps:0/572(0%)

AGG38744.1 MFLQIFVAVTLVQYVSSQCTYSSWWYSFDTPGQSKCNEINSYINALDRNDVNWADDALSN 60

MFLQIF+AVTLVQYVSSQCTYSSWWYSFDTPGQSKCN+INSYINALDRNDVNWADDALSN

A0A182YTZ4 MFLQIFLAVTLVQYVSSQCTYSSWWYSFDTPGQSKCNDINSYINALDRNDVNWADDALSN 60

AGG38744.1 LEGVQCCRPPAPWNNVEQQVVYEDWTATLDSDYTWAFCRVGYFLQGLYRSDTGWPRFKGY 120

LEGVQCCRPPAPWNNVEQQVVYEDWTATLDSDYTWAFCRVGYFLQGLYRSDTGWPRFKGY

A0A182YTZ4 LEGVQCCRPPAPWNNVEQQVVYEDWTATLDSDYTWAFCRVGYFLQGLYRSDTGWPRFKGY 120

AGG38744.1 LFNLESARCTKPANHPLNYGTCQDIDVSSCMGRKGQCSCPGGYFLTGLYRADGDDLYFLK 180

LFNLESARCTKPANHPLNYGTCQDIDVSSCMGRKGQCSCPGGYFLTGLYRADGDDLYFLK

A0A182YTZ4 LFNLESARCTKPANHPLNYGTCQDIDVSSCMGRKGQCSCPGGYFLTGLYRADGDDLYFLK 180

AGG38744.1 KIRCCTPAAKPLEMDEKSKIQTRIMDTTLWNMATLAHYMGYGWCYGCHGLAVGEDFTRNG 240

KIRCCTPAAKPLEMDEKSKIQTRIMDTTLWNMATLAHYMGYGWCYGCHGLAVGEDFTRNG

A0A182YTZ4 KIRCCTPAAKPLEMDEKSKIQTRIMDTTLWNMATLAHYMGYGWCYGCHGLAVGEDFTRNG 240

AGG38744.1 FTWAADTRTFWGKWCEGDKNGERLNLVFGDWGFAVKEIIYGKSVIEDLQAESVDSGVLYN 300

FTWAADTRTFWGKWCEGDKNGERLNLVFGDWGFAVKEIIYGKSVIEDLQAESVDSGVLYN

A0A182YTZ4 FTWAADTRTFWGKWCEGDKNGERLNLVFGDWGFAVKEIIYGKSVIEDLQAESVDSGVLYN 300

AGG38744.1 RASSPVTESIERSKTIQETITHSTTSTFTNSHGLGVELEFEIASVKGKASYKTRFEYSTS 360

RASSPVTESIERSKTIQETITHSTTSTFTNSH LGVELEFEIASVKGKASYKTRFEYSTS

A0A182YTZ4 RASSPVTESIERSKTIQETITHSTTSTFTNSHELGVELEFEIASVKGKASYKTRFEYSTS 360

AGG38744.1 TTNSKSISETQGFTKQSSITLGPMEGAKYEVIMSKSRTTVPYTAIITTKFSTEMKGFLRW 420

TTNSKSISETQGFTKQSSITLGPMEGAKYEVIMSKSRTTVPYTAIITTKFSTEMKGFLRW

A0A182YTZ4 TTNSKSISETQGFTKQSSITLGPMEGAKYEVIMSKSRTTVPYTAIITTKFSTEMKGFLRW 420

AGG38744.1 EDGNGNFHQDYRTNSGRPTFNYRFGDSSVPFYKALKKQSDNNEGVWMWGMLFQKFPDARR 480

EDGNGNFHQDYRTNSGRPTFNYRFGDSSVPFYKALKKQSDNNEGVWMWGMLFQKFPDARR

A0A182YTZ4 EDGNGNFHQDYRTNSGRPTFNYRFGDSSVPFYKALKKQSDNNEGVWMWGMLFQKFPDARR 480

AGG38744.1 VTNRLTDETQYQFTLAGKLEKVEGTSVNVKWEKMKLNRRDVSGNDEPGSNITTYIAASGP 540

V NRLTDETQYQFTL GKLEKVEGTSVNVKWEKMKLNRRDVSGND PGSNITTYIAASGP

A0A182YTZ4 VINRLTDETQYQFTLTGKLEKVEGTSVNVKWEKMKLNRRDVSGNDAPGSNITTYIAASGP 540

AGG38744.1 ADKPAVVEYPKVNLNNKEPFKPIEIPVTEVKV 572

ADKPAVVEYPKVNLNNKEPFKPIEI VTEVKV

A0A182YTZ4 ADKPAVVEYPKVNLNNKEPFKPIEISVTEVKV 572

**C:**

CLUSTAL O (1.2.4) multiple sequence alignment

A0A182YTN9 MLVQFLFAATLLQYVSSQCTYSSWWYSFDTPGQSKCNEINSYINALDRNDVNWADDALSN 60

AGG38744.1 MFLQIFVAVTLVQYVSSQCTYSSWWYSFDTPGQSKCNEINSYINALDRNDVNWADDALSN 60

A0A182YTZ4 MFLQIFLAVTLVQYVSSQCTYSSWWYSFDTPGQSKCNDINSYINALDRNDVNWADDALSN 60

*::*::.*.**:*************************:**********************

A0A182YTN9 LEGVQCCRPPAPWNNVEQQVVYEDWTATLDSDYTWAFCRVGYFLQGLYRSDTGWPRFKGY 120

AGG38744.1 LEGVQCCRPPAPWNNVEQQVVYEDWTATLDSDYTWAFCRVGYFLQGLYRSDTGWPRFKGY 120

A0A182YTZ4 LEGVQCCRPPAPWNNVEQQVVYEDWTATLDSDYTWAFCRVGYFLQGLYRSDTGWPRFKGY 120

************************************************************

A0A182YTN9 LFNLESARCTKPANHPLNYGNCQDIDVSSCMGRKGQCSCPGGYFLTGLYRADGDDLYFLK 180

AGG38744.1 LFNLESARCTKPANHPLNYGTCQDIDVSSCMGRKGQCSCPGGYFLTGLYRADGDDLYFLK 180

A0A182YTZ4 LFNLESARCTKPANHPLNYGTCQDIDVSSCMGRKGQCSCPGGYFLTGLYRADGDDLYFLK 180

********************.***************************************

A0A182YTN9 KIRCCTPAAKPLEMDEKSKIQTRIMDTTLWNMATLAHYLGYGWCYGCRGVAVGEDFTRNG 240

AGG38744.1 KIRCCTPAAKPLEMDEKSKIQTRIMDTTLWNMATLAHYMGYGWCYGCHGLAVGEDFTRNG 240

A0A182YTZ4 KIRCCTPAAKPLEMDEKSKIQTRIMDTTLWNMATLAHYMGYGWCYGCHGLAVGEDFTRNG 240

**************************************:********:*:**********

A0A182YTN9 FTWAADTRSFWGKWCEGDKNGERLNLVFGDWGFAVKEIIYGKSVIEDLQAESVDSGVLYN 300

AGG38744.1 FTWAADTRTFWGKWCEGDKNGERLNLVFGDWGFAVKEIIYGKSVIEDLQAESVDSGVLYN 300

A0A182YTZ4 FTWAADTRTFWGKWCEGDKNGERLNLVFGDWGFAVKEIIYGKSVIEDLQAESVDSGVLYN 300

********:***************************************************

A0A182YTN9 RASSPVTESIDRTKTIEETVTHSTTSTFTNSHELGIELNFEIASVSGKASYTTKFEYSKA 360

AGG38744.1 RASSPVTESIERSKTIQETITHSTTSTFTNSHGLGVELEFEIASVKGKASYKTRFEYSTS 360

A0A182YTZ4 RASSPVTESIERSKTIQETITHSTTSTFTNSHELGVELEFEIASVKGKASYKTRFEYSTS 360

**********:*:***:**:************ **:**:******.*****.*:****.:

A0A182YTN9 TTNEKSISQTAGFTKKSSITLGPMEGAKYEIIMSKSRTTVPYTAIITTKFSTEMKGFLRW 420

AGG38744.1 TTNSKSISETQGFTKQSSITLGPMEGAKYEVIMSKSRTTVPYTAIITTKFSTEMKGFLRW 420

A0A182YTZ4 TTNSKSISETQGFTKQSSITLGPMEGAKYEVIMSKSRTTVPYTAIITTKFSTEMKGFLRW 420

***.****:* ****:**************:*****************************

A0A182YTN9 EDGNGNFHQDYRTNSGRPTYNYRFGDSSVPFYKALKKQSDNNEGVWMWGMLFQKFPDARR 480

AGG38744.1 EDGNGNFHQDYRTNSGRPTFNYRFGDSSVPFYKALKKQSDNNEGVWMWGMLFQKFPDARR 480

A0A182YTZ4 EDGNGNFHQDYRTNSGRPTFNYRFGDSSVPFYKALKKQSDNNEGVWMWGMLFQKFPDARR 480

*******************:****************************************

A0A182YTN9 VINRLTDETQYQFTLTGKLEKVEGTSVNVKWEKIKLNRRDVSGNDAPGSNITTYIAASGP 540

AGG38744.1 VTNRLTDETQYQFTLAGKLEKVEGTSVNVKWEKMKLNRRDVSGNDEPGSNITTYIAASGP 540

A0A182YTZ4 VINRLTDETQYQFTLTGKLEKVEGTSVNVKWEKMKLNRRDVSGNDAPGSNITTYIAASGP 540

* *************:*****************:*********** **************

A0A182YTN9 ADKPAVVEYPKVNLNNKEPFKPIEIPVTEVKV 572

AGG38744.1 ADKPAVVEYPKVNLNNKEPFKPIEIPVTEVKV 572

A0A182YTZ4 ADKPAVVEYPKVNLNNKEPFKPIEISVTEVKV 572

************************* ******

**Figure 1—figure supplement 4. The two Biomphalysin variants (UniProtKB/TrEMBL: A0A182YTN9 and A0A182YTZ4) identified by LC-MS/MS were different from the previously published Biomphalysin (GenBank: AGG38744.1).**
