# Supplementary material for: Coordination of humoral immune factors dictates compatibility between Schistosoma mansoni and Biomphalaria glabrata
Source: eLife. 2020 Jan 9;9:e51708. doi: 10.7554/eLife.51708 (PMC6970513; doi:10.7554/eLife.51708)
Supplement: Figure 1—source data 4. — (A) The amino acid sequence information of protein A0A2C9L9F5 derived from the B. glabrata proteome database (Genome Accession: GCA_000457365). Peptides identified by LC-MS/MS are highlighted in gray. (B) Alignment of multiple BgFREP amino acid sequences and distribution of identified peptides. Peptides identified by LC-MS/MS are highlighted in gray. The GenBank accession numbers of each entry are: BgMFREP1 partial, AAK13549; BgMFREP2 precursor, AAK13550; BgFREP3-2 precursor, AAK28656; BgMFREP4 precursor, AAK13551; BgMFREP5 partial, AAK13546; BgMFREP6 partial, AAK13552; BgFREP7.1 precursor, AAK28657; BgMFREP8 partial, AAK13553; BgMFREP9 partial, AAK13554; BgMFREP10 partial, AAK13555; BgMFREP11 partial, AAK13556; BgFREP12.1 precursor, AAO59918; BgFREP12-FBG2, AAT58639; BgFREP13.1 precursor, AAO59922 and BgFREP14, ABO61860. (C) Blast analysis of protein A0A2C9L9F5 in NCBI database. The top 30 proteins with the highest scores are listed. (D) Multiple sequence alignment of 30 BgFREP2 family proteins listed in C. Peptides identified by LC-MS/MS are highlighted in gray. [file elife-51708-fig1-data4.docx]

**A:**

>tr|A0A2C9L9F5|A0A2C9L9F5_BIOGL Fibrinogen C-terminal domain-containing protein OS=Biomphalaria glabrata OX=6526 PE=4 SV=1

MASLPLRLVLLVSMATLIRSSSWLNFTGNSETIRELIQPLKLTCTFQISKNDSENDSQVLFMSIYHETKRVIASISKYQPVATSLYPSVTKVQGQIYHSNESKDSHLQVTWTHPKLSESGKYFCLAHAWNSTSQNSVFDADITVNVIKSSTDDLAVALSYIQDRLDKDGVDSIQISRTSPTLPESCRDVISSEDRVVVTLASGLKVMCDTKTDGGGWIIFQRRINGNVDFYRSWKEYRDGFGSFSIGEFYLGNENIFKLTSRKKYDLRIDLEFNNTKYFALYTRFEILGEQDYYKLQIGGYSGNAGDALTNIHNDKFFSTYDKDNDLDTSGSCAVAYKGAWWYRDCYDSNLNGKWGSDRINWSKLTGVTKSVTFTEMKIREIELD

**B:**

CLUSTAL O (1.2.4) multiple sequence alignment

*Bg*MFREP1 PARTIAL ------------------------------------------------------------ 0

*Bg*MFREP2 PRE ------------------------------------------------------------ 0

*Bg*FREP3-2 PRE MARLFLLFILCVFVVSLAGSELVIDVQPNVISPEITPQLVINCSITNNEVQTLDLIKSLT 60

*Bg*MFREP4 PRE ------------------------------------------------------------ 0

*Bg*MFREP5 PARTIAL ------------------------------------------------------------ 0

*Bg*MFREP6 PARTIAL ------------------------------------------------------------ 0

*Bg*FREP7.1 PRE MTNLLLRLVFFQSLLPLLSSELVIDVQPDIISAEITAQLVINCSVTNNQVQHLDVIRSLT 60

*Bg*MFREP8 PARTIAL ------------------------------------------------------------ 0

*Bg*MFREP9 PARTIAL ------------------------------------------------------------ 0

*Bg*MFREP10PARTIAL ------------------------------------------------------------ 0

*Bg*MFREP11PARTIAL ------------------------------------------------------------ 0

*Bg*FREP12.1 PRE -MTLLQHFLLFVSLFLFSSSELVIDVQPNVISAEITAQLVINCSITNNQVQHLDVIRSLT 59

*Bg*FREP12-FBG2 ------------------------------------------------------------ 0

*Bg*FREP13.1 PRE -MATFLYVILVVALVTLSTSELVIDVQPNVISPEITAQLAINCSVTNNQAQNIDVIKSLT 59

*Bg*FREP14 ------------------------------------------------------------ 0

*Bg*MFREP1 PARTIAL ------------------------------------------------------------ 0

*Bg*MFREP2 PRE ------------------------------------------------------------ 0

*Bg*FREP3-2 PRE LSRYNETIREFDELIALDSLTLNLKQFVRFKYSQISFGNRYITLILHNPTQFDARIYKCN 120

*Bg*MFREP4 PRE ------------------------------------------------------------ 0

*Bg*MFREP5 PARTIAL ------------------------------------------------------------ 0

*Bg*MFREP6 PARTIAL ------------------------------------------------------------ 0

*Bg*FREP7.1 PRE LSRYNQTLRDFEDITALDLLTLNLKQLVKFKHSHISFGNVFISLTLLYPTQFDANVYRCS 120

*Bg*MFREP8 PARTIAL ------------------------------------------------------------ 0

*Bg*MFREP9 PARTIAL ------------------------------------------------------------ 0

*Bg*MFREP10PARTIAL ------------------------------------------------------------ 0

*Bg*MFREP11PARTIAL ------------------------------------------------------------ 0

*Bg*FREP12.1 PRE LSRYDETLKEFLDLITLEAKTLNLSQLVQLHHAQISFGNLSISLTLHNPTQFDAKVYRCK 119

*Bg*FREP12-FBG2 ------------------------------------------------------------ 0

*Bg*FREP13.1 PRE LSRYNETIRDFEVMIXLDLLTLNLKQLVQFNYSLISFGNVFISLTILHPTKSDAKVYRCS 119

*Bg*FREP14 ------------------------------------------------------------ 0

*Bg*MFREP1 PARTIAL ------------------------------------------------------------ 0

*Bg*MFREP2 PRE ------------------------MASLPLR--------LVLLV----SMATLIRSSSWL 24

*Bg*FREP3-2 PRE ATGTNSEGANISLFAKKAVEYETNSTALIEEIRRIKK--DENYCSFKKDDLSDSKQRSRV 178

*Bg*MFREP4 PRE -------------------------------------MKNLLLCLF----LVSATLGSRL 19

*Bg*MFREP5 PARTIAL ------------------------------------------------------------ 0

*Bg*MFREP6 PARTIAL ------------------------------------------------------------ 0

*Bg*FREP7.1 PRE VKGGDPNNKDMSLFSKKTVEYETNSTALVEEIRRYKIDENKCLCSLASNDRTSTNKRLRV 180

*Bg*MFREP8 PARTIAL ------------------------------------------------------------ 0

*Bg*MFREP9 PARTIAL ------------------------------------------------------------ 0

*Bg*MFREP10PARTIAL ------------------------------------------------------------ 0

*Bg*MFREP11PARTIAL ------------------------------------------------------------ 0

*Bg*FREP12.1 PRE VEGDKTNAASSSIVAKKEVEYRTNMTALIEEIRRLKVVEKNDQCSLKXEELTSYHQKTKL 179

*Bg*FREP12-FBG2 ------------------------------------------------------------ 0

*Bg*FREP13.1 PRE VKGDNSNERNISLFAKKAVEYETNSTALIEEIQRCKKVKSKCQCSLENNDSSSNYKRSRV 179

*Bg*FREP14 ------------------------MGALIL---------HVILCAS---IVPVISSRPKL 24

*Bg*MFREP1 PARTIAL ------------------------------------------------------------ 0

*Bg*MFREP2 PRE NFTGNSETIRELIQPLKLTCTFQISKNDSDNDSQVLFMSIYHETKRVIASISKYQPVATS 84

*Bg*FREP3-2 PRE YFSGSSDIIKERIEPLTLKCTFQVLKTDQNETSRLQSLYILHESKGVIAYVNKDQPVVTS 238

*Bg*MFREP4 PRE SFNANVEKINEVIXPLMLTCSFEVSRNDSWQNTKVQLMYIMHETKGFVATITKDQNITG- 78

*Bg*MFREP5 PARTIAL -------------------------------------------SNRIIANINKDRQVLTP 17

*Bg*MFREP6 PARTIAL ------------------------------------------------------------ 0

*Bg*FREP7.1 PRE NFSGNSEIIKERVETLTLNCTFQVLNQDQNETSSLQSLYILHETNGVIANINKGQPVLK- 239

*Bg*MFREP8 PARTIAL ------------------------------------------------------------ 0

*Bg*MFREP9 PARTIAL ------------------------------------------------------------ 0

*Bg*MFREP10PARTIAL ------------------------------------------------------------ 0

*Bg*MFREP11PARTIAL ------------------------------------------------------------ 0

*Bg*FREP12.1 PRE HFVGSSKVIKELIEPLTLTCSLQNLDN----NSTVQFMYILHESNGVIATINKDQPVVTT 235

*Bg*FREP12-FBG2 ------------------------------------------------------------ 0

*Bg*FREP13.1 PRE HFSGSSEIIKERIETVTLNCTYQALKHQRNE-TSLQSLYILHEANGVIANINKGQPVVQ- 237

*Bg*FREP14 KFSGNAEILKELIEPLILSCSLKSFNNETNEHFKVHIMFIQHETNGVISTLSKDQAVAVS 84

*Bg*MFREP1 PARTIAL ------------------------------------------------------------ 0

*Bg*MFREP2 PRE LYPSVTKVQGHIYHSNESKDSYLQVTWTHPKLSESGKYFCLAHAWNSTSQNSVFDADITV 144

*Bg*FREP3-2 PRE LQGSNIQDVEGEIYDNAIKDSYLQVTWSNLKHSESGKYFCEAHNKYSEGRIDKSSNMLTI 298

*Bg*MFREP4 PRE NADMTFSEGQGTLNNEIDNTSFXQVTWKNASNELSGKYICVVHATNAEGKVEFLSASLKV 138

*Bg*MFREP5 PARTIAL SQRDNSKNIQGEIHDDGSKDSYLQVTWSNVKSSESGKYFCEANVKHSDGRAERLSEMLII 77

*Bg*MFREP6 PARTIAL ------------------------------------------------------------ 0

*Bg*FREP7.1 PRE --GSHLKDAQGNIFDSGLKDSYLQVTWSNVKLSDSGKYFCEANVKHSDGRAERLSEMLIL 297

*Bg*MFREP8 PARTIAL ------------------------------------------------------------ 0

*Bg*MFREP9 PARTIAL ------------------------------------------------------------ 0

*Bg*MFREP10PARTIAL ------------------------------------------------------------ 0

*Bg*MFREP11PARTIAL ------------------------------------------------------------ 0

*Bg*FREP12.1 PRE KQESNFNTAKGVLSDTKSKASFIEVSWSYIKSSESGNYFCGAHVMGPDGRSERLNEMLAI 295

*Bg*FREP12-FBG2 ------------------------------------------------------------ 0

*Bg*FREP13.1 PRE --GSNLKNAEGEIFHNESKDSYLQVTWSNLKFSESGKYFCEANVKHSDGRAERLSEMLII 295

*Bg*FREP14 A-DQSSTHAHGKIYNKDLQDSYLQVILKNPKISESGKYFCLAYAKNSTGQDSVFQSTVTI 143

*Bg*MFREP1 PARTIAL ------------------------------------------------------------ 0

*Bg*MFREP2 PRE NVIKSSTDDLAVALSYIQDRLDKD------------------------------------ 168

*Bg*FREP3-2 PRE TVERPTFDDLVEAMHKLFTQVDGAKESLKAINQNIKNINKDLDFKEQNITSIKQEVIRNQ 358

*Bg*MFREP4 PRE QVQKLEIADLAQYVVDLTARVKESDDKIQNYTR--------------NVTSI-------- 176

*Bg*MFREP5 PARTIAL TVVRPTFDDLVKVVEKLLEQFNEYKTHLQENV---EKNRAMLDRNKQTILLIKKDVLANQ 134

*Bg*MFREP6 PARTIAL ------------------------------------------------------------ 0

*Bg*FREP7.1 PRE TVVSPTVDDLVKVIEKLLGQVDEDTKHIQENKQNIKNIKEELKTKEQNILSNTADLNSTQ 357

*Bg*MFREP8 PARTIAL ------------------------------------------------------------ 0

*Bg*MFREP9 PARTIAL ------------------------------------------------------------ 0

*Bg*MFREP10PARTIAL ------------------------------------------------------------ 0

*Bg*MFREP11PARTIAL ------------------------------------------------------------ 0

*Bg*FREP12.1 PRE TVSNPTFDDVVKVIPKLLRQADIEKENILENKQNIYDIKEYFKSKQQNIISIKDGLNTNR 355

*Bg*FREP12-FBG2 ------------------------------------------------------------ 0

*Bg*FREP13.1 PRE TVVSPTFDDLVKVIEKLLGQVDGDTRHIQENNQSIKNIKEELKIKEQNIISITADLNSTQ 355

*Bg*FREP14 KVLKPKADDLVQVLGQLLKRVDTLEQLLKGNETNFGGV---------------------- 181

*Bg*MFREP1 PARTIAL ------------------------------------------------------------ 0

*Bg*MFREP2 PRE ------------------------------------------------------------ 168

*Bg*FREP3-2 PRE NNIQILSEDLNIKEQNLTSIKADLSTKQQTFLNIKED----------------------- 395

*Bg*MFREP4 PRE ------------------------------------------------------------ 176

*Bg*MFREP5 PARTIAL QSLQNMKEDWNSNQT--------------------------------------------- 149

*Bg*MFREP6 PARTIAL ------------------------------------------------------------ 0

*Bg*FREP7.1 PRE QTIRSMKEDIAINQHNMSSLKEFVDANLESLQSIKEDLNIQQRNIIS------------- 404

*Bg*MFREP8 PARTIAL ------------------------------------------------------------ 0

*Bg*MFREP9 PARTIAL ------------------------------------------------------------ 0

*Bg*MFREP10PARTIAL ------------------------------------------------------------ 0

*Bg*MFREP11PARTIAL ------------------------------------------------------------ 0

*Bg*FREP12.1 PRE HNIKSIADDLNVNKENIASHNDEINTLRQMVNNVQSDLSICKKSIHNFSNDLDTKKQSIA 415

*Bg*FREP12-FBG2 ------------------------------------------------------------ 0

*Bg*FREP13.1 PRE QIISIIKEDITQNQQNISSMKEDLIINQEN------------------------------ 385

*Bg*FREP14 ------------------------------------------------------------ 181

*Bg*MFREP1 PARTIAL ------------------------------------------------------------ 0

*Bg*MFREP2 PRE ------------------------------------------------------------ 168

*Bg*FREP3-2 PRE -----------------------------------------------VILNQQIIHKIKQ 408

*Bg*MFREP4 PRE ----------------------------------------------------------KE 178

*Bg*MFREP5 PARTIAL -----------------------------------------------------NIISIKE 156

*Bg*MFREP6 PARTIAL ------------------------------------------------------------ 0

*Bg*FREP7.1 PRE ---------------VKEDIAINQQNISSIKTDVAVNEENLINLQKDFNIQQRNISSIKE 449

*Bg*MFREP8 PARTIAL ------------------------------------------------------------ 0

*Bg*MFREP9 PARTIAL ------------------------------------------------------------ 0

*Bg*MFREP10PARTIAL ------------------------------------------------------------ 0

*Bg*MFREP11PARTIAL ------------------------------------------------------------ 0

*Bg*FREP12.1 PRE SHKDELNSFGQIVNSLKDDLRTNKQNLQSVTDEENTNKENINQLKEDFKSNKQKIQNITQ 475

*Bg*FREP12-FBG2 ------------------------------------------------------------ 0

*Bg*FREP13.1 PRE ----------------------------------------LKNVKEDFNIQQRNILSLEK 405

*Bg*FREP14 ------------------------------------------------------------ 181

*Bg*MFREP1 PARTIAL -------------------------------------------DVRGYNVITNRQPVSCR 17

*Bg*MFREP2 PRE -----------------------------------------GVDSIQISRASPTLPESCR 187

*Bg*FREP3-2 PRE DLNTYRHN-----------MSNIEEHLEVILTNLSTASIKVKNQTDEGSKMSYPPRKSCR 457

*Bg*MFREP4 PRE ELNALKENHLAA------------------LRS--LDIIKKVNKNLQLSCECLAKPTSCR 218

*Bg*MFREP5 PARTIAL ELQTHRQN-----------MSTLKENFETVFSNFSTALIDIKNQIVK-ERSGFKQVLSCR 204

*Bg*MFREP6 PARTIAL -----------------------------------------GVDSIQISRASPTLPESCR 19

*Bg*FREP7.1 PRE DFKTFYKNT--------------SSFQEIILANLSATLEKVKN------ETELLHPTSCR 489

*Bg*MFREP8 PARTIAL ------------------------------------------------------------ 0

*Bg*MFREP9 PARTIAL ------------------------------------------------------------ 0

*Bg*MFREP10PARTIAL ------------------------------------------------------------ 0

*Bg*MFREP11PARTIAL ------------------------------------------------------------ 0

*Bg*FREP12.1 PRE EVNANRQNIINLNNTSQQSLSKLEADLETQLTNLSTALTQIN------EKIKKGLPSSCR 529

*Bg*FREP12-FBG2 ------------------------------------------------------------ 0

*Bg*FREP13.1 PRE DFHTHQQN-----------ISNFQENLEIVLSNFSTALMEVKNQTDK-ERKGDDQITSCR 453

*Bg*FREP14 ----------------------------------TA-------DDILISTTHKTPFTSCR 200

*Bg*MFREP1 PARTIAL DVNSTEDRMVVTLASGLKVMCDTKTDGGGWIIFQRRINGKVDFYRGWKEYRDGFGDYNIG 77

*Bg*MFREP2 PRE DVISSEDRVVVTLASGLKVMCDTKTDGGGWIIFQRRINGYVDFYRGWKEYRDGFGDYDIG 247

*Bg*FREP3-2 PRE DVNSTDERVVVTLTSGLKVMCDTKTDGGGWIIFQRRINGNVDFYRGWKEYRDGFGDYNIG 517

*Bg*MFREP4 PRE DVISTEDRVVVTLASGLEVMCDTTTDGGGWTIFQRRFNGSIDFYRDWKEYRDGFGDYNIG 278

*Bg*MFREP5 PARTIAL DVRSIADRLVVFLISGLKVMCDTKTDGGGWLIFQRRINGKVDFYRGWKEYRDGFGDYDIG 264

*Bg*MFREP6 PARTIAL DVISSEDRVVVTLASGSKVMCDTKTDGGGWIIFRRRINGNVDFYRGWKEYRDGFGSFSIG 79

*Bg*FREP7.1 PRE KVIYKEDRAIVTLASGLKVMCDTKTDGGGWIIFQRRVNGSVDFYRGWQEYRDGFGDYNIG 549

*Bg*MFREP8 PARTIAL ----------------------------GWTMFQRRITGNLSFYRGWEEYKYGFGDVTTG 32

*Bg*MFREP9 PARTIAL ----------------------------GWIIFQRRINGSVDFYRGWKEYRDGFGDYNIG 32

*Bg*MFREP10PARTIAL ----------------------------GWTIFQRRINGKVDFYRGWKEYRDGFGDYNIG 32

*Bg*MFREP11PARTIAL ----------------------------GWIIFQRRINGKVDFYRGWKEYRDGFGDYTIG 32

*Bg*FREP12.1 PRE EINSFQERVIVTLTSGLKVMCDTKTDGGGWIIFQRRINGKVDFYRGWKEYRDGFGDYDIG 589

*Bg*FREP12-FBG2 ----------------------TKTDGGGWIIFQRRINGKVNFYRGWKEYRDGFGDYDIG 38

*Bg*FREP13.1 PRE DVTSKDDRVVVTLASGLKVMCDTKTDGGGWIIFQRRINGKVDFYRNWQVYRDGFGDYDIG 513

*Bg*FREP14 DVNSSDERVVVTLASEQKVMCDTKTDGGGWIIIQRRIMGYVDFYRGWKEYRDGFGDYNIG 260

** :::**. * :.***.*: *: ***. *

*Bg*MFREP1 PARTIAL EFYLGNENIFMLTSGRQYELRIDMEFKTKKYFAKYSRFKVLSEANNYQLKIESFSGNAGD 137

*Bg*MFREP2 PRE EFYLGNENIFKLTSSKKYDLRIDLEFNNTKYFAFYTRFEILGEQDYYKLQIGGYSGNAGD 307

*Bg*FREP3-2 PRE EFYLGNENIYMLTSTGQYNLRIDLKYKNKAFFAQYSGFKILSEKEKYKLNIGAYSGNSGD 577

*Bg*MFREP4 PRE EFYLGNENIFNLTSSRKYELRFDLEYENKKYFAHYSDFKLLDENNKYKLIIGSYSGNAGD 338

*Bg*MFREP5 PARTIAL EFYLGNENIFNLTSTGKYDLRIDLKYND-FYHAQYSDFQILSEKEKYKLKIGAYSGNAGD 323

*Bg*MFREP6 PARTIAL EFYLGNENIFKLTSSKKYDLRIDLEFNNTKYFALYTSFEILXXQDYYTLQIGGYSGNAGD 139

*Bg*FREP7.1 PRE EFYLGNENIFKLTSTGKYNLRIDLVLKNQPYYAQYSIFQILSEKNHYKINVGGYSGNAGN 609

*Bg*MFREP8 PARTIAL EFYLGNENMHQITTKRRHELQIDLTFNQAKFSATYSRFSLYGEPEKYRLKVSGYSGNAGD 92

*Bg*MFREP9 PARTIAL EFYLGNENIFNLTSTGQYDLRIDLEYNDKKYFAQYENFKVLSETEKYKLKIRKYSGNAGN 92

*Bg*MFREP10PARTIAL EFYLGNEKVYQLTSEKENELRVDLETNITSYFAYYSEFYLLNETENYKLKVGGFNGTMSD 92

*Bg*MFREP11PARTIAL EFYLGNEYISKLTSTRNFDLRIDFKFNHKTYFVEFSDFRILNETNNYQLKIGKYKGNASD 92

*Bg*FREP12.1 PRE EFYLGNENIFGLISTGQYDLRIDLEFNNTNYFAQYENFKVLSETEKYKLQTGTYSGNAGD 649

*Bg*FREP12-FBG2 EFYLGNENIFKLTSTGQYDLRIDLEFKNTKYFAQYEDFKVLSETEKYKLQIGDYLGNAGD 98

*Bg*FREP13.1 PRE EFYLGNENVYNLTTSGNFDLRIDLKFNYQSYFAQYTHFKLLSEKEFYRLKFGDYSGNAGD 573

*Bg*FREP14 EFYLGNENIHQLTSKKPHELRIDLEFDNENYFAQYSSFLLLGEEEFYKLQIGDYSGNAGD 320

******* : : : :*:.*: . : . : * : : * : : *. .:

*Bg*MFREP1 PARTIAL SLT-YHNDQFFSTF---------------------------------------------- 150

*Bg*MFREP2 PRE ALTNIHNDKFFSTYDKDNDLGTSGSCAVTHKGAWWYR-DCYDSNLNGKWGSDR----VNW 362

*Bg*FREP3-2 PRE NFS-SHNNAFFTTFDRDNDEYS-YNCAVDYTGAWWYHSSCLNCNLNGKWGSSDFAKGVNW 635

*Bg*MFREP4 PRE SMR-RHVNKFFTTFDKDNDDSPNDNCAIIRRGAWWYQ-NCADVNLNGNWGRGE-PDGVFW 395

*Bg*MFREP5 PARTIAL SLS-YHNDAHFSTYDKDNDNSS-INCASTVSGAWWYK-SCHHVNLNGRWRSKESGKSVIW 380

*Bg*MFREP6 PARTIAL ALTNIHNDKFFSTYDKDNDMDTSGSCAVDYKGAWWYR-SCYDSNLNGKWGSDW----VNW 194

*Bg*FREP7.1 PRE SLS-YHNDMYFSTFDNDNDAYSGVNCAVKYHGAWWYK-TCYESNLNGKWGSSERDKDLNW 667

*Bg*MFREP8 PARTIAL NLRKFDNVKFTT------------------------------------------------ 104

*Bg*MFREP9 PARTIAL GLF-YHNNMFFTT----------------------------------------------- 104

*Bg*MFREP10PARTIAL DLH-HNNNKAFST----------------------------------------------- 104

*Bg*MFREP11PARTIAL DFS-YHNNMQFST----------------------------------------------- 104

*Bg*FREP12.1 PRE DLS-PHNNKFFSTFDRDNDISSSTNCAEYNSGAWWYE-SCHHSNLNGQWGRTS-NKGMNW 706

*Bg*FREP12-FBG2 DLS-PHNNMFFSTFDRDNDVDSHLNCAEYCS----------------------------- 128

*Bg*FREP13.1 PRE SLS-YHKDMYFSTFDKDNNIDS-RNCATEYWGAWWYR-SCHYSHLNGVWGGKD-PKGLIW 629

*Bg*FREP14 SLA-YQKNMSFSTYDRDNDNASGENCAVSYSGAWWYN-ACHMSNLNGDWGNDAYGKGVNW 378

: . :

*Bg*MFREP1 PARTIAL ---------------------------- 150

*Bg*MFREP2 PRE SKLTG-ITKSVTFTEMKIREIELN---- 385

*Bg*FREP3-2 PRE YDLSR-FDSSVSFTEMKIREI------- 655

*Bg*MFREP4 PRE DNITV--WESVSFSEIKIREIDKEKNKS 421

*Bg*MFREP5 PARTIAL RALTG-EQESVLYSEMKIREKD------ 401

*Bg*MFREP6 PARTIAL SKLTG-ITKSVTFSEMKIREIELD---- 217

*Bg*FREP7.1 PRE NTLLQTAHVGVSFTEMKIRERE------ 689

*Bg*MFREP8 PARTIAL ---------------------------- 104

*Bg*MFREP9 PARTIAL ---------------------------- 104

*Bg*MFREP10PARTIAL ---------------------------- 104

*Bg*MFREP11PARTIAL ---------------------------- 104

*Bg*FREP12.1 PRE FKLTR-GSNSVSFTEMKIREREKNYFH- 732

*Bg*FREP12-FBG2 ---------------------------- 128

*Bg*FREP13.1 PRE DKVTN-YEASVSFTEMKIREKS------ 650

*Bg*FREP14 DGVTG-LHDSVVFSEMKLRELD------ 399

**C:**

RID: W1CY61B9014

Job Title:Protein Sequence

Program: BLASTP

Query: None ID: lcl|Query_10451(amino acid) Length: 385

Database: nr All non-redundant GenBank CDS translations+PDB+SwissProt+PIR+PRF excluding environmental samples from WGS projects

Sequences producing significant alignments:

Max Total Query E Per.

Description Score Score cover Value Ident Accession

fibrinogen-like protein A precursor [Biomphalaria glabrata] 771 771 100% 0.0 96.62 NP_001298226.1

fibrinogen-related protein 2.18 [Biomphalaria glabrata] 768 768 100% 0.0 96.36 ADK11400.1

fibrinogen-related protein 2.1 [Biomphalaria glabrata] 768 768 100% 0.0 96.36 ADE45330.1

fibrinogen-related protein 2.3 [Biomphalaria glabrata] 768 768 100% 0.0 96.10 ADE45335.1

fibrinogen-related protein 2.12 [Biomphalaria glabrata] 767 767 100% 0.0 96.10 ADK11394.1

fibrinogen-related protein 2.20 [Biomphalaria glabrata] 767 767 100% 0.0 96.10 ADK11402.1

fibrinogen-related protein 2.4 [Biomphalaria glabrata] 766 766 100% 0.0 96.10 ADE45336.1

fibrinogen-related protein 2.2 [Biomphalaria glabrata] 766 766 100% 0.0 96.10 ADE45334.1

fibrinogen-related protein 2.7 [Biomphalaria glabrata] 766 766 100% 0.0 96.10 ADE45331.1

fibrinogen-related protein 2.14 [Biomphalaria glabrata] 766 766 100% 0.0 96.10 ADK11396.1

fibrinogen-related protein 2.15 [Biomphalaria glabrata] 766 766 100% 0.0 96.10 ADK11397.1

fibrinogen-related protein 2.19 [Biomphalaria glabrata] 766 766 100% 0.0 96.10 ADK11401.1

fibrinogen-related protein 2.11 [Biomphalaria glabrata] 765 765 100% 0.0 96.10 ADK11393.1

fibrinogen-related protein 2.25 [Biomphalaria glabrata] 765 765 100% 0.0 96.10 ADK11407.1

fibrinogen-related protein 2.21 [Biomphalaria glabrata] 765 765 100% 0.0 96.10 ADK11403.1

fibrinogen-related protein 2.6 [Biomphalaria glabrata] 765 765 100% 0.0 96.10 ADE45338.1

fibrinogen-related protein 2.24 [Biomphalaria glabrata] 764 764 100% 0.0 95.84 ADK11406.1

fibrinogen-related protein 2.13 [Biomphalaria glabrata] 764 764 100% 0.0 95.84 ADK11395.1

fibrinogen-related protein 2.26 [Biomphalaria glabrata] 764 764 100% 0.0 95.84 ADK11408.1

fibrinogen-related protein 2.28 [Biomphalaria glabrata] 764 764 100% 0.0 95.84 ADK11410.1

fibrinogen-related protein 2.17 [Biomphalaria glabrata] 764 764 100% 0.0 95.58 ADK11399.1

BgMFREP2 precursor [Biomphalaria glabrata] 764 764 100% 0.0 95.58 AAK13550.1

fibrinogen-related protein 2.16 [Biomphalaria glabrata] 763 763 100% 0.0 95.58 ADK11398.1

fibrinogen-related protein 2.5 [Biomphalaria glabrata] 763 763 100% 0.0 95.84 ADE45337.1

fibrinogen-related protein 2.30 [Biomphalaria glabrata] 762 762 100% 0.0 95.58 ADK11412.1

fibrinogen-related protein 2.8 [Biomphalaria glabrata] 762 762 100% 0.0 95.84 ADK11390.1

fibrinogen-related protein 2.27 [Biomphalaria glabrata] 761 761 100% 0.0 95.84 ADK11409.1

fibrinogen-related protein 2.29 [Biomphalaria glabrata] 761 761 100% 0.0 95.58 ADK11411.1

fibrinogen-related protein 2.22 [Biomphalaria glabrata] 760 760 100% 0.0 95.84 ADK11404.1

fibrinogen-related protein 2.9 [Biomphalaria glabrata] 758 758 100% 0.0 95.58 ADK11391.1

**D:**

CLUSTAL O(1.2.4) multiple sequence alignment

ADK11409.1 MASLPLRLVLLVSMATLIRSSSWLNFTGNSETIRELIQPLKLTCTFQISKNDSENDSQVL 60

AAK13550.1 MASLPLRLVLLVSMATLIRSSSWLNFTGNSETIRELIQPLKLTCTFQISKNDSDNDSQVL 60

AAC47700.1 -------------MATLIRSSSWLNFTGNSETIRELIQPLKLTCTFQISKNDSDNDSQVL 47

ADK11411.1 MASLPLRLVLLVSMATLIRSSSWLNFTGNSEAIRELIQPLKLTCTFQISKNDSENDSQVL 60

ADK11412.1 MASLPLRLVLLVSMATLIRSSSWLNFTGNSETIRELIQPLKLTCTFQISKNDSENDSQVL 60

ADK11398.1 MASLPLRLVLLVSMATLIRSSSWLNFTGNSETIRELIQPLKLTCTFQISKNDSENDSQVL 60

ADK11399.1 MASLPLRLVLLVSMATLIRSSSWLNFTGNSETIRELIQPLKLTCTFQISKNDSENDSQVL 60

ADK11391.1 MASLPLRLVLLVSMATLIRSSSWLNFTGNSETIRELIQPLKLTRTFQISKNDSENDSQVL 60

ADK11404.1 MASLPLRLVLLVSMATLIRSSSWLNFTGNSETIRELIQPLKLTCTFQISKNDSENDSQVL 60

ADE45337.1 MASLPLRLVLLVSMATLIRSSSWLNFTGNSETIRELIQPLKLTCTFQISKNDSENDSQVL 60

ADK11410.1 MASLPLRLVLLVSMVTLIRSSSWLNFTGNSETIRELIQPLKLTCTFQISKNDSENDSQVL 60

ADK11408.1 MASLPLRLVLLVSMATLIRSSSWLNFTGNSETIRELIQPLKLTCTFQISKNDSENDSQVL 60

ADK11395.1 MASLPLRLVLLVSMATLIRSSSWLNFTGNSETIRELIQPLKLTCTFQISKNDSESDSQVL 60

ADK11406.1 MASLPLRLVLLVSMATLIRSSSWLNFTGNSETIRELIQPLKLTCTFQISKNDSENDSQVL 60

ADK11390.1 MASLPLRLVLLVSMATLIRSSSWLNFTGNSETIRELIQPLKLTCTFQISKNDSENDSQVL 60

ADE45338.1 MASLPLRLVLLVSMATLIRSSSWLNFTGNSETIRELIQPLKLTCTFQISKNDSENDSQVL 60

ADK11403.1 MASLPLRLVLLVSMATLIRSSSWLNFTGNSETIRELIQPLKLTCTFQISKNDSENDSQVL 60

ADK11407.1 MASLPLRLVLLVSMATLIRSSSWLNFTGNSETIRELIQPLKLTCTFQISKNDSENDSQVL 60

ADK11393.1 MASLPLRLVLLVSMATLIRSSSWLNFTGNSETIRELIQPLKLTCTFQISKNDSENDSQVL 60

ADK11401.1 MASLPLRLVLLVSMATLIRSSSWLNFTGNSETIRELIQPLKLTCTFQISKNDSENDSQVL 60

ADK11397.1 MASLPLRLVLLVSMATLIRSSSWLNFTGNSETIRELIQPLKLTCTFQISKNDSENDSQVL 60

ADE45331.1 MASLPLRLVLLVSMATLIRSSSWLNFTGNSETIRELIQPLKLTCTFQISKNDSENDSQVL 60

ADE45334.1 MASLPLRLVLLVSMATLIRSSPWLNFTGNSETIRELIQPLKLTCTFQISKNDSENDSQVL 60

ADE45336.1 MASLPLRLVLLVSMATLIRSSSWLNFTGNSETIRELIQPLKLTCTFQISKNDSENDSQVL 60

ADK11402.1 MASLPLRLVLLVSMATLIRSSSWLNFTGNSETIRELIQPLKLTCTFQISKNDSENDSQVL 60

ADK11394.1 MASLPLRLVLLVSMATLIRSSSWLNFTGNSETIRELIQPLKLTCTFQISKNDSENDSQVL 60

ADE45335.1 MASLPLRLVLLVSMATLIRSSSWLNFTGNSETIRELIQPLKLTCTFQISKNDSENDSQVL 60

ADK11400.1 MASLPLRLVLLVSMATLIRSSSWLNFTGNSETIRELIQPLKLTCTFQISKNDSENDSQVL 60

NP_001298226.1 MASLPLRLVLLVSMATLIRSSSWLNFTGNSETIRELIQPLKLTCTFQISKNDSENDSQVL 60

ADE45330.1 MASLPLRLVLLVSMATLIRSSSWLNFTGNSETIRELIQPLKLTCTFQISKNDSENDSQVL 60

*.****** *********:*********** *********:.*****

ADK11409.1 FMSIYHETKRVIASISKYQPVATSLYPSVTKVQGQIYHSNESKDSYLQVTWTHPKLSESG 120

AAK13550.1 FMSIYHETKRVIASISKYQPVATSLYPSVTKVQGHIYHSNESKDSYLQVTWTHPKLSESG 120

AAC47700.1 FMSIYHETKRVIASISKYQPVATSLYPSVTKVQGHIYHSNESKDSYLQVTWTHPKLSESG 107

ADK11411.1 FMSIYHETKRVIASISKYQPVATSLYPSVTKVQGQIYHSNESKDSYLQVTWTHPKLSESG 120

ADK11412.1 FMSIYHETKRVIASISKYQPVATSLYPSVTKVQGQIYHSNESKDSYLQVTWTHPKLSESG 120

ADK11398.1 FMSIYHETKRVIASISKYQPVATSLYPSVTKVQGQIYHSNESKDSYLQVTWTHPKLSESG 120

ADK11399.1 FMSIYHETKRVIASISKYQPVATSLYPSVTKVQGQIYHSNESRDSYLQVTWTHPKLSESG 120

ADK11391.1 FMSIYHETKRVIASISKYQPVATSLYPSVTKVQGQIYHSNESKDSYLQVTWTHPKLSESG 120

ADK11404.1 FMSIYHETKRVIASISKYQPVATSLYPSVTKVQGQIYHSNESKDSYLQVTWTHPKLSESG 120

ADE45337.1 FMSIYHETKRVIASISKYQPVATSLYPSVTKVQGQIYHSNESKDSYLQVTWTHPKLSESG 120

ADK11410.1 FMSIYHETKRVIASISKYQPVATSLYPSVTKVQGQIYHSNESKDSYLQVTWTHPKLSESG 120

ADK11408.1 FMSIYHETKRVIASISKYQPVATSLYPSVTKVQGQIYHSNESKDSYLQVTWTHPKLSESG 120

ADK11395.1 FMSIYHETKRVIASISKHQPVATSLYPSVTKVQGQIYHSNESKDSYLQVTWTHPKLSESG 120

ADK11406.1 FMSIYHETKRVIASISKYQPVATSLYPSVTKVQGQIYHSNESKDSYLQVTWTHPKLSESG 120

ADK11390.1 FMSIYHETKRVIASISKYQPVATSLYPSVTKVQGQIYHSNESKDSYLQVTWTHPKLSESG 120

ADE45338.1 FMSIYHETKRVIASISKYQPVATSLYPSVTKVQGQIYHSNESKDSYLQVTWTHPKLSESG 120

ADK11403.1 FMSIYHETKRVIASISKYQPVATSLYPSVTKVQGQIYHSNESKDSYLQVTWTRPKLSESG 120

ADK11407.1 FMSIYHETKRVIASISKYQPVATSLYPSVTKVQGQIYHSNESKDSYLQVTWTHPKLSESG 120

ADK11393.1 FMSIYHETKRVIASISKYQPVATSLYPSVTKVQGQIYHSNESKDSYLQVTWTHPKLSESG 120

ADK11401.1 FMSIYHETKRVIASISKYQPVATSLYPSVTKVQGQIYHSNESKDSYLQVTWTHPKLSESG 120

ADK11397.1 FMSIYHETKRVIASISKYQPVATSLYPSVTKVQGQIYHSNESKDSYLQVTWTHPKLSESG 120

ADE45331.1 FMSIYHETKRVITSISKYQPVATSLYPSVTKVQGQIYHSNESKDSYLQVTWTHPKLSESG 120

ADE45334.1 FMSIYHETKRVIASISKYQPVATSLYPSVTKVQGQIYHSNESKDSYLQVTWTHPKLSESG 120

ADE45336.1 FMSIYHETKRVIASISKYQPVATSLYPSVTKVQGQIYHSNESKDSYLQVTWTHPKLSESG 120

ADK11402.1 FMSIYHETKRVIASISKYQPVATSLYPSVTKVQGQIYHSNESKDSYLQVTWTHPKLSESG 120

ADK11394.1 FMSIYHETKRVIASISKYQPVATSLYPSVTKVQGQIYHSNESKDSYLQVTWTHPKLSESG 120

ADE45335.1 FMSIYHETKRVIASISKYQPVATSLYPSVTKVQGQIYHSNESKDSYLQVTWTHPKLSESG 120

ADK11400.1 FMSIYHETKRVIASISKYQPVATSLYPSVTKVQGQIYHSNESKDSYLQVTWTHPKLSESG 120

NP_001298226.1 FMSIYHETKRVIASISKYQPVATSLYPSVTKVQGQIYHSNESKDSHLQVTWTHPKLSESG 120

ADE45330.1 FMSIYHETKRVIASISKYQPVATSLYPSVTKVQGQIYHSNESKDSYLQVTWTHPKLSESG 120

************:****:****************:*******:**:******:*******

ADK11409.1 KYFCLAHAWNSTSQNSVFDADITVNVIKSSTDDLAVALSYIQDRLDKDGVDSIQISRASP 180

AAK13550.1 KYFCLAHAWNSTSQNSVFDADITVNVIKSSTDDLAVALSYIQDRLDKDGVDSIQISRASP 180

AAC47700.1 KYFCLAHAWNSTSQNSVFDADITVNVIKSSTDDLAVALSYIQDRLDKDGVDSIQISRASP 167

ADK11411.1 KYFCLAHAWNSTSQNSVFDADITVNVIKSSTDDLAVALSYIQDRLDKDGVDSIQISRASP 180

ADK11412.1 KYFCLAHAWNSTSQNSVFDADITVNVTKSSTNDLAVALSYIQDRLDKDGVDSIQISRASP 180

ADK11398.1 KYFCLAHAWNSTSQNSVFDADITVNVIKSSTDDLAVALSYIQDRLDKDGVDSIQISRASP 180

ADK11399.1 KYFCLAHAWNSTSQNSVFDADITVNVIKSSTDDLVVALSYMQDRLDKDGVDSIQISRASP 180

ADK11391.1 KYFCLAHAWNSTSQNSVFDADITVNVIKSSTDDLAVALSYIQDRLDKDGVDSIQISRASP 180

ADK11404.1 KYFCLAHAWNSTSQNSVFDADITVNVIKSSTDDLAVALSYIQDRLDKDGVDSIQISRASP 180

ADE45337.1 KYFCLAHAWNSTSQNSVFDADITVNVIKSSTDDLAVALSYIQDRLDKDGVDSIQISRASP 180

ADK11410.1 KYFCLAHAWNSTSQNSVFDADITVNVIKSSTDDLAVALSYIQDRLDKDGVDSIQISRASP 180

ADK11408.1 EYFCLAHAWNSTSQNSVFDADITVNVIKSSTDDLAVALSYIQDRLDKDGVDSIQISRASP 180

ADK11395.1 KYFCLAHAWNSTSQNSVFDADITVNVIKSSTDDLAVALSYIQDRLDKDGVDSIQISRASP 180

ADK11406.1 KYFCLAHAWNSTSQNSVFDADITVNVIKSSTDDLAVALSYIQDRLDKDGVDSIQISRASP 180

ADK11390.1 KYFCLAHAWNSTSQNSVFDADITVNVIKSSTDDLAVALSYIQDRLDKDGVDSIQISRASP 180

ADE45338.1 KYFCLAHAWNSTSQNSVFDADITVNVIKSSTDDLAVALSYIQDRLDKDGVDSIQISRASP 180

ADK11403.1 KYFCLAHAWNSTSQNSVFDADITVNVIKSSTDDLAVALSYIQDRLDKDGVDSIQISRASP 180

ADK11407.1 KYFCLAHAWNSTSQNSVFDADITVNVIKSSTDDLAVALSYIQDRLDKGGVDSIQISRASP 180

ADK11393.1 KYFCLAHAWNSTSQNSVFDADITVNVIKSSTDDLAVALSYIQDRLDKDGVDSIQISRASP 180

ADK11401.1 KYFCLAHAWNSTSQNSVFDADITVNVIKSSTDDLAVALSYIQDRLDKDGVDSIQISRACP 180

ADK11397.1 KYFCLAHAWNSTSQNSVFDADITVNVIKSSTDDLAVALSYIQDRLDKDGVDSIQISRASP 180

ADE45331.1 KYFCLAHAWNSTSQNSVFDADITVNVIKSSTDDLAVALSYIQDRLDKDGVDSIQISRASP 180

ADE45334.1 KYFCLAHAWNSTSQNSVFDADITVNVIKSSTDDLAVALSYIQDRLDKDGVDSIQISRASP 180

ADE45336.1 KYFCLAHAWNSTSQNSVFDADITVNVIKSSTDDLAVALSYIQDRLDKDGVDSIQISRASP 180

ADK11402.1 KYFCLAHAWNSTSQNSVFDADITVNVIKSSTDDLAVALSYIQDRLDKDGVDSIQISRASP 180

ADK11394.1 KYFCLAHAWNSTSQNSVFDADITVNVIKSSTDDLAVALSYIQDRLDKDGVDSIQISRASP 180

ADE45335.1 KYFCLAHAWNSTSQNSVFDADITVNVIKSSTDDLAVALSYIQDRLDKDGVDSIQISRASP 180

ADK11400.1 KYFCLAHAWNSTSQNSVFDADITVNVIKSSTDDLAVALSYIQDRLDKDGVDSIQISRVSP 180

NP_001298226.1 KYFCLAHAWNSTSQNSVFDADITVNVIKSSTDDLAVALSYIQDRLDKDGVDSIQISRASP 180

ADE45330.1 KYFCLAHAWNSTSQNSVFDADITVNVIKSSTDDLAVALSYIQDRLDKDGVDSIQISRASP 180

:************************* ****:**.*****:******.*********..*

ADK11409.1 TLPESCRDVISSEDRVVVTLASGLKVMCDTKTDGGGWIIFQRRINGYVDFYRGWKEYRDG 240

AAK13550.1 TLPESCRDVISSEDRVVVTLASGLKVMCDTKTDGGGWIIFQRRINGYVDFYRGWKEYRDG 240

AAC47700.1 TLPESCRDVISSEDRVVVTLASGLKVMCDTKTDGGGWIIFQRRINGYVDFYRGWKEYRDG 227

ADK11411.1 TLPESCRDVISSEDRVVVTLASGVKVMCDTKTDGGGWIIFQRRINGYVDFYRGWKEYRDG 240

ADK11412.1 TLPESCRDVISSEDRVVVTLASGLKVMCDTKTDGGGWIIFQRRINGYVDFYRGWKEYRDG 240

ADK11398.1 TLPESCRDVISSEDRVVVTLASGLKVMCDTKTDGGGWIIFQRRINGYVDFYRGWKEYRDG 240

ADK11399.1 TLPESCRDVISSEDRVVVTLASGLKVMCDTKTDGGGWIIFQRRINGYVDFYRGWKEYRDG 240

ADK11391.1 TLPESCRDVISSEDRVVVTLASGLKVMCDTKTDGGGWIIFQRRINGYVDFYRGWKEYRDG 240

ADK11404.1 TLPESCRDVISSEDRVVVTLASGLKVTCDTKTDGGGWIIFQRRINGYVDFYRGWKEYRDG 240

ADE45337.1 TLPESCRDVISSEDRVVVTLASGLKVMCDTKTDGGGWIIFQRRINGYVDFYRGWKEYRDG 240

ADK11410.1 TLPESCRDVISSEDRVVVTLASGLKVMCDTKTDGGGWIIFQRRINGYVDFYRGWKEYRDG 240

ADK11408.1 TLPESCRDVISSEDRVVVTLASGLKVMCDTKTDGGGWIIFQRRINGYVDFYRGWKEYRDG 240

ADK11395.1 TLPESCRDVISSEDRVVVTLASGLKVMCDTKTDGGGWIIFQRRINGYVDFYRGWKEYRDG 240

ADK11406.1 TLPESCRDVISSEDRVVVTLASGLKVMCDTKTDGGGWIIFQRRINGYVDFYRGWKEYRDG 240

ADK11390.1 TLPESCRDVISSEDRVVVTLASGLKVMCDTKTDGGGWIIFQRRINGYVDFYRGWKEYRDG 240

ADE45338.1 TLPESCRDVISSEDRVVVTLASGLKVMCDTKTDGGGWIIFQRRINGYVDFYRGWKEYRDG 240

ADK11403.1 TLPESCRDVISSEDRVVVTLASGLKVMCDTKTDGGGWIIFQRRINGYVDFYRGWKEYRDG 240

ADK11407.1 TLPESCRDVISSEDRVVVTLASGLKVMCDTKTDGGGWIIFQRRINGYVDFYRGWKEYRDG 240

ADK11393.1 TLPESCRDVISSEDRVVVTLASGLKVMCDTKTDGGGWIIFQRRINGYVDFYRGWKEYRDG 240

ADK11401.1 TLPESCRDVISSEDRVVVTLASGLKVMCDTKTDGGGWIIFQRRINGYVDFYRGWKEYRDG 240

ADK11397.1 TLPESCRDVISSEDRVVVTLASGLKVMCDTKTDGGGWIIFQRRINGYVDFYRGWKEYRDG 240

ADE45331.1 TLPESCRDVISSEDRVVVTLASGLKVMCDTKTDGGGWIIFQRRINGYVDFYRGWKEYRDG 240

ADE45334.1 TLPESCRDVISSEDRVVVTLASGLKVMCDTKTDGGGWIIFQRRINGYVDFYRGWKEYRDG 240

ADE45336.1 TLPESCRDVISSEDRVVVTLASGLKVMCDTKTDGGGWIIFQRRINGYVDFYRGWKEYRDG 240

ADK11402.1 TLPESCRDVISSEDRVVVTLASGLKVMCDTKTDGGGWIIFQRRINGYVDFYRGWKEYRDG 240

ADK11394.1 TLPESCRDVISSEDRVVVTLASGLKVMCDTKTDGGGWIIFQRRINGYVDFYRGWKEYRDG 240

ADE45335.1 TLPESCRDIISSEDRVVVTLASGLKVMCDTKTDGGGWIIFQRRINGYVDFYRGWKEYRDG 240

ADK11400.1 TLPESCRDVISSEDRVVVTLASGLKVMCDTKTDGGGWIIFQRRINGYVDFYRGWKEYRDG 240

NP_001298226.1 TLPESCRDVISSEDRVVVTLASGLKVMCDTKTDGGGWIIFQRRINGYVDFYRGWKEYRDG 240

ADE45330.1 TLPESCRDVISSEDRVVVTLASGLKVMCDTKTDGGGWIIFQRRINGYVDFYRGWKEYRDG 240

********:**************:** *********************************

ADK11409.1 FGDYDIGEFYLGNENIFKLTSSKKYDLRIDLEFNNTKYFAFYTRFEILGEQDYYKLQIGG 300

AAK13550.1 FGDYDIGEFYLGNENIFKLTSSKKYDLRIDLEFNNTKYFAFYTRFEILGEQDYYKLQIGG 300

AAC47700.1 FGDYDIGEFYLGNENIFKLTSSKKYDLRIDLEFNNTKYFAFYTRFEILGEQDYYKLQIGG 287

ADK11411.1 FGDYDIGEFYLGNENIFKLTSSKKYDLRIDLEFNNTKYFAFYTRFEILGEQDYYKLQIGG 300

ADK11412.1 FGDYDIGEFYLGNENIFKLTSSKKYDLRIDLEFNNTKYFAFYTRFEILGEQDYYKLQIGG 300

ADK11398.1 FGDYDIGEFYLGNENIFKLTFSKKYDLRIDLEFNNTKYFAFFTRFEILGEQDYYKLQIGG 300

ADK11399.1 FGDYDIGEFYLGNENIFKLTSSKKYDLRIDLEFNNTKYFAFYTRFEILGEQDYYKLQIGG 300

ADK11391.1 FGDYDIGEFYLGNENIFKLTSSKKYDLRIDLEFNNTKYFAFYTRFEILGEQDYYKLQIGG 300

ADK11404.1 FGDYDIGEFYLGNENIFKLTSSKKYDLRIDLEFNNTKYFAFYTRFEILGEQDYYKLQIGG 300

ADE45337.1 FGDYDIGEFYLGNENIFKLTSSKKYDLRIDLEFNNTKYFAFYTRFEILGEQDYYKLQIGG 300

ADK11410.1 FGDYDIGEFYLGNENIFKLTSSKKYDLRIDLEFNNTKYFAFYTRFEILGEQDYYKLQIGG 300

ADK11408.1 FGDYDIGEFYLGNENIFKLTSSKKYDLRIDLEFNNTKYFAFYTRFEILGEQDYYKLQIGG 300

ADK11395.1 FGDYDIGEFYLGNENIFKLTSSKKYDLRIDLEFNNTKYFAFYTRFEILGEQDYYKLQIGG 300

ADK11406.1 FGDYDIGEFYLGNENIFKLTSSKKYDLRIDLEFNNTKYFAFYTRFEILGEQDYYKLQTGG 300

ADK11390.1 FGDYDIGEFYLGNENIFKLTSSKKYDLRIDLEFNNTKYFAFSTRFEILGEQDYYKLQIGG 300

ADE45338.1 FGDYDIGEFYLGNENIFKLTSSKKYDLRIDLEFNNTKYFAFYTRFEILGEQDYYKLQIGG 300

ADK11403.1 FGDYDIGEFYLGNENIFKLTSSKKYDLRIDLEFNNTKYFAFYTRFEILGEQDYYKLQIGG 300

ADK11407.1 FGDYDIGEFYLGNENIFKLTSSKKYDLRIDLEFNNTKYFAFYTRFEILGEQDYYKLQIGG 300

ADK11393.1 FGDYDIGEFYLGNENIFKLTSSKKYDLRIDLEFNNTKYFAFYTRFEILGEQDYYKLQIGG 300

ADK11401.1 FGDYDIGEFYLGNENIFKLTSSKKYDLRIDLEFNNTKYFAFYTRFEILGEQDYYKLQIGG 300

ADK11397.1 FGDYDIGEFYLGNENIFKLTSSKKYDLRIDLEFSNTKYFAFYTRFEILGEQDYYKLQIGG 300

ADE45331.1 FGDYDIGEFYLGNENIFKLTSSKKYDLRIDLEFNNTKYFAFYTRFEILGEQDYYKLQIGG 300

ADE45334.1 FGDYDIGEFYLGNENIFKLTSSKKYDLRIDLEFNNTKYFAFYTRFEILGEQDYYKLQIGG 300

ADE45336.1 FGDYDIGEFYLGNENIFKLTSSKKYDLRIDLEFNNTKYFAFYTRFEILGEQDYYKLQIGG 300

ADK11402.1 FGDYDIGEFYLGNENIFKLTSSKKYDLRIDLEFNNTKYFAFYTRFEMLGEQDYYKLQIGG 300

ADK11394.1 FGDYDIGEFYLGNENIFKLTSSKKYDLRIDLEFNNTKYFAFYTRFEVLGEQDYYKLQIGG 300

ADE45335.1 FGDYDIGEFYLGNENIFKLTSSKKYDLRIDLEFNNTKYFAFYTRFEILGEQDYYKLQIGG 300

ADK11400.1 FGDYDIGEFYLGNENIFKLTSSKKYDLRIDLEFNNTKYFAFYTRFEILGEQDYYKLQIGG 300

NP_001298226.1 FGDYDIGEFYLGNENIFKLTSSKKYDLRIDLEFNNTKYFAFYTRFEILGEQDYYKLQIGG 300

ADE45330.1 FGDYDIGEFYLGNENIFKLTSSKKYDLRIDLEFNNTKYFAFYTRFEILGEQDYYKLQIGG 300

******************** ************.******* ****:********** **

ADK11409.1 YSGNAGDALTNTHNDKFFSTYDKDNDLGTSDSCAVTYKGAWWYRDCCDSNLNGKWGSDRI 360

AAK13550.1 YSGNAGDALTNIHNDKFFSTYDKDNDLGTSGSCAVTHKGAWWYRDCYDSNLNGKWGSDRV 360

AAC47700.1 YSGNAGDALTNIHNDKFFSTYDKDNDLGTSGSCAVTHKGAWWYRDCYDSNLNGKWGSDRV 347

ADK11411.1 YSGNAGDALTNIHNDKFFSTYDKDNDLGTSGSYAVTHKGAWWYRDCYDSNLNGKWGSDRI 360

ADK11412.1 YSGNAGDALTNIHNDKFFSTYDKDNDLGTSGSCAVTHKGAWWYRDCYDSNLNGKWGSDRT 360

ADK11398.1 YSGNAGDALTNIHNDKFFSTYDKDNDLGTSGSCAVTHKGAWWYRDCYDSNLNGKWGSDRI 360

ADK11399.1 YSGNAGDALTNIHNDKFFSTYDKDNDLGTSGSCAVTHKGAWWYRDCYDSNLNGKWGSDRI 360

ADK11391.1 YSGNAGDALTNIHNDKFFSTYDKDNDLGTSGSCAVTHKGAWWYRDCYDSNLNGKWGSDRI 360

ADK11404.1 YSGNAGDALTNIHNDKFFSTYDKDNDLGTSGSCAVTHKGAWWYRDCYDSNLNGKRGSDRI 360

ADE45337.1 YSGNAGDALTNIHNDKFFSTYDKDNDLGTSGSCAVTHKGAWWYRDCYDSNLNGKWGSDRI 360

ADK11410.1 YSGNAGDALTNIHNDKFFSTYDKDNDLGTSGSCAVTHKGAWWYRDCYDSSLNGKWGSDRI 360

ADK11408.1 YSGNAGDALTNIHNDKFFSTYDKDNDLGTSGSCTVTHKGAWWYRDCYDSNLNGKWGSDRI 360

ADK11395.1 YSGNAGDALTNIHNDKFFSTYDKDNDLGTSGSCAVTHKGAWWYRDCYDSNLNGKWGSDRI 360

ADK11406.1 YSGNAGDALANIHNDKFFSTYDKDNDLGTSGSCAVTHKGAWWYRDCYDSNLNGKWGSDRI 360

ADK11390.1 YSGNAGDALTNIHNDKFFSTYDKDNDLGTSGSCAVTHKGAWWYRDCYDSNLNGKWGSDRI 360

ADE45338.1 YSGNAGDALTNIHNDKFFSTYDKDNDLGTSGSCAVTHKGAWWYRDCYDSNLNGKWGSDRI 360

ADK11403.1 YSGNAGDALTNIHNDKFFSTYDKDNDLGTSGSCAVTHKGAWWYRDCYDSNLNGKWGSDRI 360

ADK11407.1 YSGNAGDALTNIHNDKFFSTYDKDNDLGTSGSCAVTHKGAWWYRDCYDSNLNGKWGSDRI 360

ADK11393.1 YSGNAGDALTNIHNDKFFSTYDKDNDLGTSGSCAVTHKGAWWYRDCYDSNLNGKWGSDRI 360

ADK11401.1 YSGNAGDALTNIHNDKFFSTYDKDNDLGTSGSCAVTHKGAWWYRDCYDSNLNGKWGSDRI 360

ADK11397.1 YSGNAGDALTNIHNDKFFSTYDKDNDLGTSGSCAVTHKGAWWYRDCYDSNLNGKWGSDRI 360

ADE45331.1 YSGNAGDALTNIHNDKFFSTYDKDNDLGTSGSCAVTHKGAWWYRDCYDSNLNGKWGSDRI 360

ADE45334.1 YSGNAGDALTNIHNDKFFSTYDKDNDLGTSGSCAVTHKGAWWYRDCYDSNLNGKWGSDRI 360

ADE45336.1 YSGNAGDALTNIHNDKFFSTYDKDNDLGTSGSCAVTHKGAWWYRDCYDSNLDGKWGSDRI 360

ADK11402.1 YSGNAGDALTNIHNDKFFSTYDKDNDLGTSGSCAVTHKGAWWYRDCYDSNLNGKWGSDRI 360

ADK11394.1 YSGNAGDALTNIHNDKFFSTYDKDNDLGTSGSCAVTHKGAWWYRDCYDSNLNGKWGSDRI 360

ADE45335.1 YSGNAGDALTNIHNDKFFSTYDKDNDLGTSGSCAVTHKGAWWYRDCYDSNLNGKWGSDRI 360

ADK11400.1 YSGNAGDALTNIHNDKFFSTYDKDNDLGTSGSCAVTHKGAWWYRDCYDSNLNGKWGSDRI 360

NP_001298226.1 YSGNAGDALTNIHNDKFFSTYDKDNDLGTSGSCAVTHKGAWWYRDCYDSNLNGKWGSDRI 360

ADE45330.1 YSGNAGDALTNIHNDKFFSTYDKDNDLGTSGSCAVTHKGAWWYRDCYDSNLNGKWGSDRI 360

*********:* ******************.* :**:********* **.*:** ****

ADK11409.1 NWSKLTGITKSVTFTEMKIREIELN 385

AAK13550.1 NWSKLTGITKSVTFTEMKIREIELN 385

AAC47700.1 NWSKLTGITKSVTFTEMKIREIELN 372

ADK11411.1 NWSKLTGITKSVTFTEMKIREIELN 385

ADK11412.1 NWSKLTGITKSVTFTEMKIREIELN 385

ADK11398.1 NWSKLTGITKSITFTEMKIREIELN 385

ADK11399.1 NWSKLTGITKSVTFTEMKIREIELN 385

ADK11391.1 NWSKLTGITKSVTFRGMKIREIELN 385

ADK11404.1 NWSKLTGITKSVTFTEMKIREIELN 385

ADE45337.1 NWSKLTGITKSVTFTEMKFRGIELN 385

ADK11410.1 NWSKLTGITKSVTFTEMKIREIELN 385

ADK11408.1 NWSKLTGITKSVTFTEMKIREIELN 385

ADK11395.1 NWSKLTGITKSVTFTEMKIREIELN 385

ADK11406.1 NWSKLTGITKSVTFTEMKIREIELN 385

ADK11390.1 NWSKLTGITKSVTFREMKIREIELN 385

ADE45338.1 NWSKLTGITKSVTFTGMKIREIELN 385

ADK11403.1 NWSKLTGITKSVTFTEMKIREIELN 385

ADK11407.1 NWSKLTGITKSVTFTEMKIREIELN 385

ADK11393.1 NWSKLTGITKSVTFREMKIREIELN 385

ADK11401.1 NWSKLTGITKSVTFTEMKIREIELN 385

ADK11397.1 NWSKLTGITKSVTFTEMKIREIELN 385

ADE45331.1 NWSKLTGITKSVTFTEMKIREIELN 385

ADE45334.1 NWSKLTGITKSVTFTEMKIREIELN 385

ADE45336.1 NWSKLTGITKSVTFTEMKIREIELN 385

ADK11402.1 NWSKLTGITKSVTFTEMKIREIELN 385

ADK11394.1 NWSKLTGITKSVTFTEMKIREIELN 385

ADE45335.1 NWSKLTGITKSVTFTEMKIREIELN 385

ADK11400.1 NWSKLTGITKSVTFTEMKIREIELN 385

NP_001298226.1 NWSKLTGITKSVTFTEMKIREIELN 385

ADE45330.1 NWSKLTGITKSVTFTEMKIREIELN 385

***********:** **:* ****

**Figure 1—figure supplement 5. The identified protein (UniProtKB/TrEMBL: A0A2C9L9F5) represented a variant of *Bg*FREP2 members that was distinct from other *Bg*FREP members, but was indistinguishable from other *Bg*FREP2 variants**.
