# Supplementary material for: Coordination of humoral immune factors dictates compatibility between Schistosoma mansoni and Biomphalaria glabrata
Source: eLife. 2020 Jan 9;9:e51708. doi: 10.7554/eLife.51708 (PMC6970513; doi:10.7554/eLife.51708)
Supplement: Figure 1—source data 5. — Peptides identified by LC-MS/MS are highlighted in gray, the overlapping region of peptides are bold underline. GenBank annotations of BgFREP3.2 (AAK28656.1) are used for locate the individual BgFREP3 domains: orange, signal peptide; green, IgSF1 domain; blue, small connecting region; pink, IgSF2 domain; black, ICR; red, FBG domain. [file elife-51708-fig1-data5.docx]

CLUSTAL O (1.2.4) multiple sequence alignment

*Bg*FREP3.3 AEO50747.1 --MLLSLELIRKFPTDTRSSHELVIDAQPEVISLELTPQLVVNCSITDSHVPGLDTINSL 58

*Bg*FREP3.3 AAO59915.1 MARLFLLFILC-VFVVSLAGSELVIDVQPNVISPEITPQLVINCSITNNEVQQLDLIKSL 59

*Bg*FREP3.2 AEO50746.1 MERLFLLLVLC-VFVVSLAGSELVIDVQPNVISPEITPQLVINCSITNNKVQQLDLIKSL 59

*Bg*FREP3.2 AAK28656.1 MARLFLLFILC-VFVVSLAGSELVIDVQPNVISPEITPQLVINCSITNNEVQTLDLIKSL 59

*Bg*FREP3.1 AEO50745.1 MARLFPRLVLC-VFIVPLAGSELVIDVQPNVISPEITPQLVINCSVTNNQVQHLEVIKSL 59

*Bg*MFREP3 AAK13548.1 ---LFLLFILC-VFVVSLAGSELVIDVQPNVISPEITPQLVINCSITNNEVQTLDLIKSL 56

*Bg*FREP3.3 *: :: . :. *****.**:*** *:*****:***:*:..* *: *:**

*Bg*FREP3.3 AEO50747.1 SLSRYNETKKEFDVLLSLDTHTLSLQQLVQFRHAQISFGNLYVTLTLRNPTQSDAKVYRC 118

*Bg*FREP3.3 AAO59915.1 TLSRYNETIREFDDLIALDSLTLNLKQFVRFKYSQISFGNLYITLTLPNPTQFDARIYKC 119

*Bg*FREP3.2 AEO50746.1 TLSRYNETIRDFDELIALDSLTLNLKQFVRFKYSQISFGNRYITLILHNPTQFDARIYKC 119

*Bg*FREP3.2 AAK28656.1 TLSRYNETIREFDELIALDSLTLNLKQFVRFKYSQISFGNRYITLILHNPTQFDARIYKC 119

*Bg*FREP3.1 AEO50745.1 TLSRYNEIIREFDELIALDSLTQNLKQFVRFKYSQISFGNLYITLTLPNPTQFDARIYRC 119

*Bg*MFREP3 AAK13548.1 TLSRYNETIREFDELIALDSLTLNLKQFVRFKYSQISFGNRYITLILHNPTQFDARIYKC 116

*Bg*FREP3.3 :****** ::** *::**: * .*:*:*:*:::****** *:** * **** **::*:*

*Bg*FREP3.3 AEO50747.1 NVSGDDSLWKNITRVFKKEIKYETNLTVLLEEIRRLREEKDRDQLSCQKEKLN----DSK 174

*Bg*FREP3.3 AAO59915.1 NATGANSDGTNISLFAKKAVEYETNSTALIEEIRRIKKDENY--CSFKKDDLADIKQRSR 177

*Bg*FREP3.2 AEO50746.1 NATGDNSEGANISLFAKKGVEYETNSTALIEEIRRIKKDENY--CSFKKDDLSDSKQRSR 177

*Bg*FREP3.2 AAK28656.1 NATGTNSEGANISLFAKKAVEYETNSTALIEEIRRIKKDENY--CSFKKDDLSDSKQRSR 177

*Bg*FREP3.1 AEO50745.1 NADGANSEGTNISLFTKKAVEYETNSTALIEEIRRIKKDENK--CSLKKDDLSDIKQRWR 177

*Bg*MFREP3 AAK13548.1 NATGTNSEGANISLFAKKAVEYETNSTALIEEIRRIKKDENY--CSFKKDDLSDXKQRSR 174

*Bg*FREP3.3 *. * :* **: . ** ::**** *.*:*****:::::: * :*:.* :

*Bg*FREP3.3 AEO50747.1 LHFVGNSKVVKELFDPLTLTCSIQDLMNDRNETSTVQSIYILHEANGIIATISKDQPVVT 234

*Bg*FREP3.3 AAO59915.1 VYFSGSSDIIKERIEPLTLKCTFQVLKTDQNDTSRLQSLYILHESKGVIAYVNKDQPVVT 237

*Bg*FREP3.2 AEO50746.1 VYFSGSSDIIKERIEPLTLKCTFQVLKTDQNEISRLQSLYILHETKGVIAYVNKDQPVVT 237

*Bg*FREP3.2 AAK28656.1 VYFSGSSDIIKERIEPLTLKCTFQVLKTDQNETSRLQSLYILHESKGVIAYVNKDQPVVT 237

*Bg*FREP3.1 AEO50745.1 VYFSESSKIIKERIEPLTLKCTFQILTPDENETSRLQSLYILHESNGVVANINKDQAVIT 237

*Bg*MFREP3 AAK13548.1 VYFSGSSDIIKERIEPLTLKCTFQVLKTDQNETSRLQSLYILHESKGVIAYVNKDQPVVT 234

*Bg*FREP3.3 ::* .*.::** ::****.*::* * *.*: * :**:*****::*::* :.*** *:*

*Bg*FREP3.3 AEO50747.1 TNQDVNLLAVIGKLHDDSSKNSYLQVTWSNPKFSESGKYFCGAHANNAQGRNEHWNEMLT 294

*Bg*FREP3.3 AAO59915.1 SLQGSNIQDVEGEIYDNAIKDSYLQVTWSNLKHSESGKYFCEAHNKYSEGRIDKSSNMLT 297

*Bg*FREP3.2 AEO50746.1 SLQGSHIQDVEGEIYDNAIKDSYLQVTWSNLKHTESGKYFCEAHNQYSEGRIDKTSNMLT 297

*Bg*FREP3.2 AAK28656.1 SLQGSNIQDVEGEIYDNAIKDSYLQVTWSNLKHSESGKYFCEAHNKYSEGRIDKSSNMLT 297

*Bg*FREP3.1 AEO50745.1 TIQGGNFENAQGEISGDQSKESYLQVTWSNLKHSDSGKYFCEAHVKHSKGKAERLNEMLT 297

*Bg*MFREP3 AAK13548.1 SLQGSNIQDVEGEIYDNAIKDSYLQVTWSNLKHSESGKYFCEAHNXYSEGRIDXSSNMLT 294

*Bg*FREP3.3 : *. :: . *:: .: *:********* *.::****** ** ::*: : .:***

*Bg*FREP3.3 AEO50747.1 ITVERLQFDDIVKVMYDIQRQVDEDKKRLQTFHENLTNNFIILNTNLQSIENVRRDVRTN 354

*Bg*FREP3.3 AAO59915.1 ITVERPTFDDLVEAMHKLFTQVDGAKESLKAINQNIKN---------------------- 335

*Bg*FREP3.2 AEO50746.1 ITVERPTFDDLVEAMHKLFTQVDGAKESLKAINQNIKN---------------------- 335

*Bg*FREP3.2 AAK28656.1 ITVERPTFDDLVEAMHKLFTQVDGAKESLKAINQNIKN---------------------- 335

*Bg*FREP3.1 AEO50745.1 IEVISPTIDDLMEVIQKLVTQVDGDKESLQDVKQNIMN---------------------- 335

*Bg*MFREP3 AAK13548.1 ITVERPTFDDLVEAXXKLFTQVDGAKESLKAINQNIKN---------------------- 332

*Bg*FREP3.3 * * :**:::. .: *** *: *: .::*: *

*Bg*FREP3.3 AEO50747.1 QESINGIKDELLSNKQNIVNNKKDINTAKESINVIKEELLSNKQNIVNNKRDIDTMEESI 414

*Bg*FREP3.3 AAO59915.1 --------------------INKDLDFKEQNITSIKQEVIRNQNNIQILSEDLNIKE--- 372

*Bg*FREP3.2 AEO50746.1 --------------------INKDLDFKEQNITSIKEEVIRNQNNIQILSEDSNIKE--- 372

*Bg*FREP3.2 AAK28656.1 --------------------INKDLDFKEQNITSIKQEVIRNQNNIQILSEDLNIKE--- 372

*Bg*FREP3.1 AEO50745.1 --------------------IKEDLNTKEQNIISIKEDLNTKQQSIISIKEEFKTKQ--- 372

*Bg*MFREP3 AAK13548.1 --------------------INKDLDFKEQNITSIKQEVIRNQNNIQILSEDLNIKE--- 369

*Bg*FREP3.3 ::*:: ::.* **::: :::.* ..: . :

*Bg*FREP3.3 AEO50747.1 NVIRHELLSNKQNIVNNKKDIHTTQESIIGILEELLRNQQNIVNNKKDINTTQDSIREIQ 474

*Bg*FREP3.3 AAO59915.1 ---------------------------------------QNLTSIKADLSTKQQTFLNIK 393

*Bg*FREP3.2 AEO50746.1 ---------------------------------------QNMTSIREDLSTKQQTFLNIK 393

*Bg*FREP3.2 AAK28656.1 ---------------------------------------QNLTSIKADLSTKQQTFLNIK 393

*Bg*FREP3.1 AEO50745.1 ---------------------------------------EN---IQKDVTINQQNIQKIK 390

*Bg*MFREP3 AAK13548.1 ---------------------------------------QNLTSIKADLSTKQQTFLNIK 390

*Bg*FREP3.3 :* : *:. .*:.: :*:

*Bg*FREP3.3 AEO50747.1 EELLRNQQNIENAKEELKSKQDSMKSIQDELLSNLHNMNISSIWKVLSNFSTAVMDMKDD 534

*Bg*FREP3.3 AAO59915.1 EDVILNQQIIHKIKQDLNTYRHNMSNIEE------------HLEVILANLSTASIKVKNQ 441

*Bg*FREP3.2 AEO50746.1 EDVILNQQNIDKIKQDLNTYRHNMSYIEE------------HLEVILANLSSASMKVKNQ 441

*Bg*FREP3.2 AAK28656.1 EDVILNQQIIHKIKQDLNTYRHNMSNIEE------------HLEVILTNLSTASIKVKNQ 441

*Bg*FREP3.1 AEO50745.1 EELDSKEQSMIIIREDFSACQQNISAFKE------------NIEIMFANLSTSFMQVKTQ 438

*Bg*MFREP3 AAK13548.1 EDVILNQQIIHKIKQDLNTYRHNMSNIEE------------HLEVILTNLSTASIKVKNQ 438

*Bg*FREP3.3 *:: ::* : ::::.: :..:. ::: : :::*:*:: :.:* :

*Bg*FREP3.3 AEO50747.1 IDKGKTEIKQNKSSITKLHPRSCRDVNSTDDRVVVTLASGLKVMCDTKTDGGGWIIFQRR 594

*Bg*FREP3.3 AAO59915.1 TDEGSK--------MSYPHRKSCRDVNSTEERVVVTLTSGLKVMCDTKTDGGGWIIFQRR 493

*Bg*FREP3.2 AEO50746.1 TDEGST--------LSYPPRKSCRDVNSTDERVVVTLTSGLKVMCDTKTDGGGWIIFQRR 493

*Bg*FREP3.2 AAK28656.1 TDEGSK--------MSYPPRKSCRDVNSTDERVVVTLTSGLKVMCDTKTDGGGWIIFQRR 493

*Bg*FREP3.1 AEO50745.1 INKGNQE------DQHLIPKIICRDVNSTDERVVVTLTSGLKVMCDTKTDGGGWIIFQRR 492

*Bg*MFREP3 AAK13548.1 TDEGSK--------MSYPPRKSCRXVNSTDDRVVVTLASGLKVMCDTKTDGGGWIIFQRR 490

*Bg*FREP3.3 ::*. ** ****::******:**********************

*Bg*FREP3.3 AEO50747.1 INGSVDFYRGWQEYRDGFGDYNIGEFYLGNENIYMLTSTGQYDLRIDLKYKNNSFFAQYS 654

*Bg*FREP3.3 AAO59915.1 INGSVDFYRDWKEYRDGFGDYNIGEFYLGNENIFNLTSNGQYNLRIDLKYKNNAFFAQYS 553

*Bg*FREP3.2 AEO50746.1 ISGNVDFYRGWKEYRDGFGDYNIGEFYLGNENIYMLTSTGQYDLRIDLKYKNNAFFAQYS 553

*Bg*FREP3.2 AAK28656.1 INGNVDFYRGWKEYRDGFGDYNIGEFYLGNENIYMLTSTGQYNLRIDLKYKNKAFFAQYS 553

*Bg*FREP3.1 AEO50745.1 INGKVDFYRGWKEYRDGFGDYTIGEFYLGNEYISKLTSTRNFDLRIDFKYNHKTYFVEYS 552

*Bg*MFREP3 AAK13548.1 INGSVDFYRGWKEYRDGFGDYNIGEFYLGNENIYMLTSTGQYNLRIDLKYKNKAFFAQYS 550

*Bg*FREP3.3 *.*.*****.*:*********.********* * ***. :::****:**:::::*.:**

*Bg*FREP3.3 AEO50747.1 SFKILSEKEKYKLNIGVYSGNAGDSFSRHNNSFFTTFDRDNDENSSNCAVVYTGAWWYKR 714

*Bg*FREP3.3 AAO59915.1 SFKILSEKEKYKLNIGAYSGNAGDSFSYHNNAFFTTFDRDNDEHSSNCAVEFRGGWWYHS 613

*Bg*FREP3.2 AEO50746.1 GFKILSEKEKYKLNIGVYSGNAGDSFSIHNNSFFTTFDRDNDENSSNCAVDYTGAWWYQS 613

*Bg*FREP3.2 AAK28656.1 GFKILSEKEKYKLNIGAYSGNSGDNFSSHNNAFFTTFDRDNDEYSYNCAVDYTGAWWYHS 613

*Bg*FREP3.1 AEO50745.1 DFRILNETNNYQLKIGKYKGNASDEFSYHNNMQFSTFDRDNDVDKRNCALQFTGAWWYH- 611

*Bg*MFREP3 AAK13548.1 GFKILSEKEKYKLNIGAYSGNSGDNFSSHNNAFFTTFDRDNDEYSYNCAVDYTGAWWYHS 610

*Bg*FREP3.3 .*:**.*.::*:*:** *.**:.*.** *** *:******* . ***: : *.***:

*Bg*FREP3.3 AEO50747.1 GCHHSNLNGKWGSSDHGKGVNWHVVSNFDSSVSFTEIKIREI 756

*Bg*FREP3.3 AAO59915.1 GCLHCNLNGQWGSTDHGKGVNWYDLSKYDSSLSFTEMKIREI 655

*Bg*FREP3.2 AEO50746.1 GCHSCNLNGKWGSTDYGKGVNWYTLSTFYSSLSFTEMKIREI 655

*Bg*FREP3.2 AAK28656.1 SCLNCNLNGKWGSSDFAKGVNWYDLSRFDSSVSFTEMKIREI 655

*Bg*FREP3.1 AEO50745.1 SCHYSNLNGQWGSKEYSKGANWESITGYEASVSFVEMKIRER 653

*Bg*MFREP3 AAK13548.1 SCLNCNLNGKWGSSDFAKGVNWYDLSRFDSSVSFTEMKIREI 652

*Bg*FREP3.3 .* .****:***.:..**.** :: : :*:**.*:****

**Figure 1—figure supplement 6. Alignment of multiple *Bg*FREP3 amino acid sequences and distribution of identified peptides.**
