# Supplementary material for: Coordination of humoral immune factors dictates compatibility between Schistosoma mansoni and Biomphalaria glabrata
Source: eLife. 2020 Jan 9;9:e51708. doi: 10.7554/eLife.51708 (PMC6970513; doi:10.7554/eLife.51708)
Supplement: Supplementary file 1. [file elife-51708-supp1.docx]

**Supplementary File 1** Primer list for cloning and quantitative RT-PCR.

| **Name** | **Strain** | **Protein** | **Primer sequence** | **Isolated or synthesized from:** |
| --- | --- | --- | --- | --- |
| **For cloning:** |  |  |  |  |
| *Bg*MFREP2 precursor  (AY012700.1) | M-line | AAK13550.1 | Forward:5'- CAC CAG GAG GAT CTT AGT AAT GGC GTC GC -3'  Reverse: 5'- GAC CCT TGG CGC GTT TAG CTC TAT TTC TCT -3' | M-line strain |
| *Bg*FREP3.2 precursor  (Derived from AY028461.1) | M-line | AAK28656.1 | Forward: 5'- CAC CAG GAG AAG AAA CGA AAT GGC TCG TCT CTT CTT GCT CTT C -3'  Reverse: 5'- CAT CTC AGT GAA CGA CAC GGA -3' | Synthesized by GenScript |
| *Bg*TEP1.1  (HM003907.1) | Brazil | ADE45332.1 | Forward: 5'- CAC CAT GAG AAT GAA GCT GAA T -3'  Reverse: 5'- TGG GCA ACA GTT GAG GCA -3' | M-line strain |
| **For q-PCR:** |  |  |  |  |
| *Bg*TEP1.1 |  |  | Forward: 5’-CACCAATGGCGATGTCATTTAG-3’  Reverse: 5’-CTGACTCTCTCAACAGGCTTAG-3’ |  |
| *Bg*Actin |  |  | Forward: 5’-GCTTCCACCTCTTCATCTCTT - 3’  Reverse: 5’ -GAACGTAGCTTCTGGACATCTG-3’ |  |
