# Supplementary material for: Coordination of humoral immune factors dictates compatibility between Schistosoma mansoni and Biomphalaria glabrata
Source: eLife. 2020 Jan 9;9:e51708. doi: 10.7554/eLife.51708 (PMC6970513; doi:10.7554/eLife.51708)
Supplement: Supplementary file 2. [file elife-51708-supp2.docx]

**Supplementary File 2** The identified peptides of *Bg*TEP1, Biomphalysin, *Bg*FREP2 and *Bg*FREP3.3 by LC-MS/MS.

|  | **Sequence** | **# PSMs** | **# Protein groups** | **Protein Group Accessions** | **# times identified** |
| --- | --- | --- | --- | --- | --- |
| ***Bg*TEP1:** | | | | | |
| P1 | FQVDVGLPPFALLSDTTLSGSVEAK | 2 | 1 | A0A2C9M761 | 1 |
| P2 | YTFGQPVYGLVLLQIGENVDTIDK | 4 | 2 | A0A2C9L049; A0A2C9M761 | 2 |
| P3 | KVTEISFEIK | 1 | 1 | A0A2C9M761 | 1 |
| P4 | ITAFVTEASTGIK | 1 | 1 | A0A2C9L049 | 1 |
| P5 | SYSMSNNYLQLSLLSK | 3 | 1 | A0A2C9LWE8 | 3 |
| P6 | ITSTEAIDSLAYEIR | 3 | 1 | A0A2C9LWE8 | 3 |
| P7 | SGVLELNGQR | 7 | 2 | A0A2C9LWE8; D7QYI1 | 4 |
| P8 | QGMMGAPMAMSFR | 1 | 1 | D7QYI1 | 1 |
| P9 | VFRPFFVSLTYPR | 1 | 1 | D7QYI1 | 1 |
| P10 | ENPFLTPITPGPGNQASNIQVR | 1 | 1 | D7QYI1 | 1 |
| P11 | SNMAADAIVR | 2 | 1 | D7QYI1 | 2 |
| P12 | ATNQLSEELNK | 2 | 1 | D7QYI1 | 2 |
| P13 | IIDLLNNGYQR | 11 | 2 | A0A2C9KIQ1; D7QYI1 | 3 |
| P14 | QNIDGSFNEFGK | 3 | 2 | A0A2C9KIQ1; D7QYI1 | 3 |
| P15 | SQLAQSTFEK | 2 | 1 | D7QYI1 | 1 |
| P16 | LDEALPSVR | 3 | 1 | D7QYI1 | 2 |
| P17 | KGSIVAIYFDK | 1 | 1 | D7QYI1 | 1 |
| **Biomphalysin:** | | | | | |
| P1 | CNEINSYINALDR | 6 | 2 | A0A182YTN9; A0A182YTZ4 | 5 |
| P2 | VGYFLQGLYR | 9 | 2 | A0A182YTN9; A0A182YTZ4 | 5 |
| P3 | GYLFNLESAR | 2 | 2 | A0A182YTN9; A0A182YTZ4 | 2 |
| P4 | GQCSCPGGYFLTGLYR | 5 | 2 | A0A182YTN9; A0A182YTZ4 | 4 |
| P5 | ADGDDLYFLK | 2 | 2 | A0A182YTN9; A0A182YTZ4 | 2 |
| P6 | NGFTWAADTR | 7 | 2 | A0A182YTN9; A0A182YTZ4 | 4 |
| P7 | WCEGDKNGER | 1 | 2 | A0A182YTN9; A0A182YTZ4 | 1 |
| P8 | LNLVFGDWGFAVK | 2 | 2 | A0A182YTN9; A0A182YTZ4 | 3 |
| P9 | SVIEDLQAESVDSGVLYNR | 9 | 2 | A0A182YTN9; A0A182YTZ4 | 6 |
| P10 | SSITLGPMEGAK | 1 | 2 | A0A182YTN9; A0A182YTZ4 | 1 |
| P10/2 | QSSITLGPMEGAK | 1 | 1 | A0A182YTZ4 | 1 |
| P11 | FGDSSVPFYK | 3 | 2 | A0A182YTN9; A0A182YTZ4 | 3 |
| P12 | LTDETQYQFTLTGK | 7 | 1 | A0A182YTN9; | 6 |
| P13 | LEKVEGTSVNVK | 2 | 2 | A0A182YTN9; A0A182YTZ4 | 1 |
| ***Bg*FREP2:** | | | | | |
| P1 | YQPVATSLYPSVTK | 16 | 1 | A0A2C9L9F5 | 9 |
| P2 | SSTDDLAVALSYIQDR | 5 | 1 | A0A2C9L9F5 | 5 |
| P3 | LDKDGVDSIQISR | 22 | 1 | A0A2C9L9F5 | 9 |
| P4 | TDGGGWIIFQR | 13 | 1 | A0A2C9L9F5 | 9 |
| P5 | DCYDSNLNGK | 1 | 1 | A0A2C9L9F5 | 1 |
| ***Bg*FREP3.3:** | | | | | |
| P1 | SIQDELLSNLHNMNISSIWKVLSNFSTAVMDMKDDIDK | 2 | 1 | AEO50747.1 | 1 |
| P2 | DIDTMEESINVIRHELLSNK | 1 | 1 | AEO50747.1 | 1 |
| P3 | NNSFFAQYSSFKILSEKEK | 3 | 1 | AEO50747.1 | 1 |
| P4 | NEHWNEMLTITVERLQFDDIVK | 1 | 1 | AEO50747.1 | 1 |
| P5 | KDINTTQDSIR | 2 | 1 | AEO50747.1 | 1 |
| P6 | QNIVNNKR | 2 | 1 | AEO50747.1; | 1 |
| P7 | LQFDDIVK | 1 | 2 | AEO50747.1; XP_013062145.1 | 1 |
| P8 | ESLKAINQNIKNINK | 8 | 8 | AAO59915.1; AAK28656.1; AEO50746.1; AAK13548.1; AAC47701.1; AQX34544.1; AQX34587.1; AQX34558.1. | 1 |
| P9 | AINQNIKNINKDLDFK | 2 | 8 | AAO59915.1; AAK28656.1; AEO50746.1; AAK13548.1; AAC47701.1; AQX34544.1; AQX34587.1; AQX34558.1. | 1 |

**PSMs:** peptide-to-spectrum matches.
